# A Dual‐Response DNA Origami Platform for Imaging and Treatment of Sepsis‐Associated Acute Kidney Injury

**DOI:** 10.1002/advs.202416330

**Published:** 2025-02-28

**Authors:** Yingying Zhao, Yadan Zhao, Yufan Ling, Zhiming Chen, Xiaofeng Wu, Xing Lu, Yao He, Houyu Wang, Fenglin Dong

**Affiliations:** ^1^ Department of Ultrasound the First Affiliated Hospital of Soochow University Suzhou Jiangsu 215006 China; ^2^ Suzhou Key Laboratory of Nanotechnology and Biomedicine Institute of Functional Nano and Soft Materials (FUNSOM) Jiangsu Key Laboratory for Carbon‐Based Functional Materials and Devices Soochow University Suzhou Jiangsu 215123 China; ^3^ State Key Laboratory of Radiation Medicine and Protection School of Radiation Medicine and Protection Soochow University Suzhou 215123 China; ^4^ Department of Ultrasound Children‘s Hospital of Soochow University Suzhou Jiangsu 215000 China; ^5^ Macao Translational Medicine Center Macau University of Science and Technology Taipa Macau SAR 999078 China; ^6^ Macao Institute of Materials Science and Engineering Macau University of Science and Technology Taipa Macau SAR 999078 China

**Keywords:** DNA origami, dual‐modal imaging, reactive oxygen species, sepsis‐associated acute kidney injury

## Abstract

Current diagnostics for sepsis‐associated acute kidney injury (SA‐AKI) detect kidney damage only at advanced stages, limiting opportunities for timely intervention. A DNA origami‐based nanoplatform is developed for the early diagnosis and treatment of SA‐AKI. Modified with a fluorophore (Cy5) and quencher (BHQ3), the DNA origami remains nonfluorescent under normal conditions. During SA‐AKI, elevated microRNA‐21 triggers a strand displacement reaction that restores the fluorescence signal, enabling real‐time detection. Additionally, the photoacoustic changes of BHQ3, driven by different excretion rates of the nanostructure and released DNA strands, enable dual‐mode imaging, enhancing diagnostic accuracy. Therapeutically, DNA origami scavenges reactive oxygen species and, when conjugated with the antimicrobial peptide Leucine‐Leucine‐37 (LL‐37), exhibits bactericidal effects. This combination boosts survival rates by 80% in SA‐AKI models. This dual‐response nanoplatform integrates precise imaging and targeted therapy, offering a powerful strategy for SA‐AKI management and advancing applications of DNA origami in precision nanomedicine.

## Graphical abstract

A dual‐response DNA origami platform is developed for the early diagnosis and therapeutic intervention of sepsis‐associated acute kidney injury.

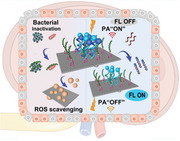

## 1 Introduction

Acute kidney injury (AKI), a syndrome defined by sudden renal dysfunction, frequently arises from sepsis in hospitalized patients, especially in intensive care unit (ICU), where it manifests as sepsis‐associated AKI (SA‐AKI).^[^ ^1^, ^2^, ^3^ ^]^ SA‐AKI is characterized by inflammation, oxidative stress, and microvascular dysfunction, among others. Nearly 50% of septic ICU patients develop AKI, with many at risk of irreversible kidney failure, necessitating early diagnosis and treatment to avoid dialysis or transplantation.^[^ ^4^, ^5^, ^6^ ^]^ Conventional diagnostics based on serum creatinine (Cre), blood urea nitrogen (BUN), and urine output detect AKI only after significant loss of renal function has occurred, delaying timely treatment.^[^ ^7^, ^8^, ^9^, ^10^ ^]^ Molecular optical probes for early AKI detection show promise,^[^ ^11^, ^12^, ^13^ ^]^ yet single‐mode imaging remains vulnerable to noise and false positives.^[^ ^14^, ^15^, ^16^, ^17^ ^]^ Dual‐mode imaging offers improved diagnostic accuracy by enhancing sensitivity and specificity.^[^ ^18^, ^19^, ^20^, ^21^ ^]^ Nanomedicine‐based therapies primarily target oxidative stress but fail to address other critical mechanisms, limiting therapeutic efficacy.^[^ ^22^, ^23^, ^24^, ^25^ ^]^ Thus, innovative strategies combining multimodal imaging and multi‐target therapies are needed to optimize both diagnosis and treatment of SA‐AKI.

Nanomaterials ≈100 nm in diameter can engage with the renal mesenchyme, migrate to proximal tubular epithelial cells, and subsequently be excreted into the proximal tubule of the kidney.^[^ ^26^, ^27^, ^28^ ^]^ These properties have motivated the design of nanosystems for diagnosing and treating renal diseases. Among various nanomaterials, DNA origami stands out as a cutting‐edge platform, offering exceptional biocompatibility, low immunogenicity, and programmability. Its precise structural control enables novel applications across different fields, including cancer therapy,^[^ ^29^, ^30^, ^31^, ^32^ ^]^ antimicrobial treatment,^[^ ^33^, ^34^ ^]^ and kidney disease management.^[^ ^25^, ^35^, ^36^, ^37^ ^]^ Particularly, rectangular DNA origami nanostructures accumulate preferentially in kidneys, guided by their compact structure and surface charge.^[^ ^25^, ^35^, ^36^, ^37^ ^]^ Beyond targeted delivery, DNA origami exhibits intrinsic antioxidant properties by scavenging reactive oxygen species (ROS) originating from the nucleophilic sites within DNA.^[^ ^25^, ^37^, ^38^, ^39^, ^40^, ^41^, ^42^ ^]^ However, the development of a DNA origami‐based platform for integrated SA‐AKI diagnosis and therapy remains an unmet need.

In this study, we present a novel nanoplatform based on rectangular DNA origami (rDON) designed for both the early diagnosis and treatment of SA‐AKI. Under physiological conditions, the quencher (BHQ3) suppresses the fluorescence signal (Cy5). However, when SA‐AKI occurs, the kidney significantly overexpresses microRNA‐21 (miR‐21). This overexpression triggers a strand displacement reaction involving the BHQ3‐modified DNA strand, leading to the recovery of the fluorescence signal. In addition to fluorescence, BHQ3's photoacoustic properties change because of the differential excretion rates of the DNA origami nanostructure and the released DNA strands. The rDON platform offers dual therapeutic benefits: its DNA origami scavenges ROS, mitigating oxidative kidney damage in SA‐AKI, while LL‐37 conjugation provides potent bactericidal activity. Caspase‐1‐cleavable linkage enhances LL‐37′s in vivo efficacy, achieving an 80% survival improvement in SA‐AKI models, surpassing single treatments.

## 2 Results and Discussion

### 2.1 Design of DNA Origami Nanoplatform for SA‐AKI Diagnosis and Therapy

**Figure 1:**
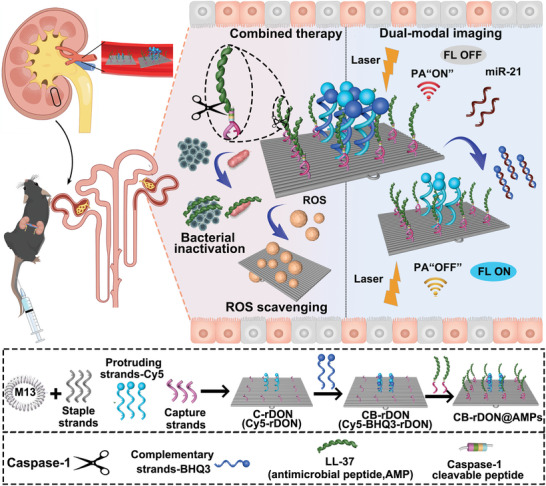
Schematic representation of a DNA origami nanoplatform designed for the early diagnosis and therapeutic intervention of SA‐AKI. Following intravenous administration, CB‐rDON@AMPs selectively accumulated within the renal tissue. In SA‐AKI mice, elevated miR‐21 triggered the detachment of BHQ3 from the DNA nanostructure, activating fluorescence while diminishing the photoacoustic signal. Simultaneously, the antimicrobial action of LL‐37, combined with the ROS‐scavenging properties of rDON, offered a synergistic approach to mitigate SA‐AKI.

We designed a DNA origami‐based nanoplatform capable of both imaging and therapeutic functions, responsive to miR‐21 and caspase‐1 for the early diagnosis and treatment of SA‐AKI (**Figure** **1**). Under normal physiological conditions, the fluorescence signal remained quenched due to Förster resonance energy transfer (FRET) between Cy5 and BHQ3,^[^ ^43^, ^44^, ^45^ ^]^ both conjugated to the DNA origami structure. However, upon the onset of SA‐AKI, miR‐21 expression significantly increased,^[^ ^46^, ^47^ ^]^ disrupting the FRET mechanism and turning the fluorescence signal “on”. Concurrently, the dissociation of BHQ3‐ssDNA from the origami nanostructure facilitated its rapid renal excretion, while the intact Cy5‐BHQ3‐rDON@antimicrobial peptides (CB‐rDON@AMPs) remained in the kidneys. This differential clearance altered the photoacoustic signal of BHQ3,^[^ ^48^, ^49^, ^50^ ^]^ allowing for dual‐modal imaging (fluorescence and photoacoustic) responsive to miR‐21 expression, thus enabling precise diagnosis of AKI. For therapeutic application, the DNA origami platform exploited its intrinsic antioxidant properties to scavenge ROS, which were abundantly produced during AKI. In addition, we functionalized the nanostructures with the antimicrobial peptide LL‐37, which has been shown to ameliorate sepsis.^[^ ^51^, ^52^, ^53^, ^54^, ^55^, ^56^ ^]^ This peptide was coupled via a caspase‐1‐cleavable peptide junction (Tyr‐Val‐Ala‐Asp, cP). This strategic linkage allowed the controlled release of LL‐37, as caspase‐1, an enzyme elevated during sepsis, cleaves the peptide linker.^[^ ^57^, ^58^, ^59^ ^]^ The linker featured a tyrosine (Tyr) end modified with N‐hydroxysuccinimide (NHS) to bind LL‐37 and an aspartic acid (Asp) end modified with 6‐maleimide to connect with SH‐ssDNA. Upon SA‐AKI occurrence, the elevated caspase‐1 levels triggered the release of LL‐37 from the DNA origami, enhancing the bactericidal and immunomodulatory effects. Collectively, this DNA origami platform provided a dual‐modal imaging approach for detecting SA‐AKI, while concurrently delivering therapeutic interventions and scavenging ROS to mitigate the effects of sepsis.

### 2.2 Preparation and Characterization of DNA Origami Nanoplatform

**Figure 2:**
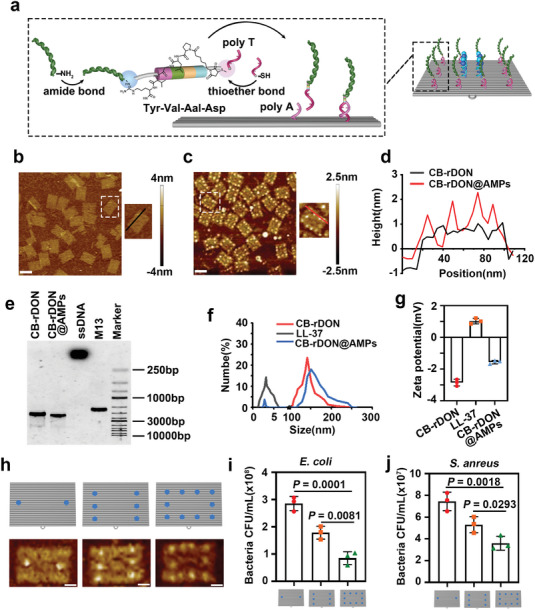
Preparation and characterization of CB‐rDON@AMPs. a) Schematic depiction of the CB‐rDON@AMPs assembly process. b,c) Representative atomic force microscopy (AFM) images of CB‐rDON and CB‐rDON@AMPs. Scale bars = 50 nm. d) Height profiles comparing CB‐rDON and CB‐rDON@AMPs. e) Agarose gel electrophoresis (AGE, 1%) analysis of CB‐rDON, CB‐rDON@AMPs, single‐stranded DNA (ssDNA), and M13mp18 genomic DNA (M13). f,g) Dynamic light scattering (DLS) (f) and zeta potential (g) analyses of CB‐rDON, LL‐37, and CB‐rDON@AMPs. All samples were ultrafiltered and dissolved in TAE/Mg^2+^ buffer prior to zeta potential measurements to remove any artifacts from residual reaction buffers. Measurements were performed in deionized water. Data were presented as mean ± standard deviation (*n* = 3). h) Representative AFM images of rDON loaded with 2, 6, and 10 peptides, respectively. Scale bars = 20 nm. i,j) Quantitative analysis of bacterial colony counts on agar plates of *E. coli* (i) and *S. aureus* (j) treated with CB‐rDON@AMPs conjugated with 2, 6, and 10 LL‐37 molecules, respectively. Data were presented as mean ± standard deviation (*n* = 3). Statistical analysis was performed using one‐way ANOVA.

The DNA origami designs are schematically shown in Figure S1 (Supporting Information), and the detailed DNA sequences are listed in Tables S1‐S4 (Supporting Information). Initially, Cy5‐rDON (C‐rDON) was constructed via a single‐step annealing method,^[^ ^60^ ^]^ where M13mp18 genomic DNA (M13)(7249 nucleotides), staple strands (≈40 nucleotides), Cy5‐ssDNA, and LL‐37 capture strands were mixed in a 1:8:8:8 molar ratio. The temperature was gradually reduced from 95 °C to ambient (Table S5, Supporting Information). The 5′ end of Cy5‐ssDNA extended from the surface of the DNA origami, and the fluorescent dye Cy5 was conjugated. Agarose gel electrophoresis (AGE) revealed that C‐rDON migrated more slowly than M13 DNA, confirming successful DNA origami assembly (Figure S2, Supporting Information). To prepare Cy5‐BHQ3‐rDON (CB‐rDON), C‐rDON was hybridized with BHQ3‐modified strands at the 3′ end and annealed for 2 h, cycling between 45 and 25 °C. BHQ3 effectively quenched Cy5 fluorescence in the 620–730 nm range, as confirmed by fluorescence spectrometry (Figure S3, Supporting Information). The quenching efficiency highlighted the successful formation of CB‐rDON. Next, LL‐37 was conjugated to DNA origami via a caspase‐1‐cleavable peptide linker (cP), as schematically illustrated in **Figure** **2**a. LL‐37 was reacted with cP at a 1:50 molar ratio for 1 hour at room temperature. The NHS group of cP conjugated to LL‐37′s amino group, while excess cP was removed via centrifugation. The purified cP‐LL‐37 complex was then reacted overnight with SH‐ssDNA, forming cP‐LL‐37‐SH‐ssDNA through maleimide‐sulfhydryl condensation. Excess SH‐ssDNA was removed using a 3 kDa filter. The final CB‐rDON@AMPs nanosheets were assembled by heating CB‐rDON with LL‐37‐conjugated DNA strands to 37 °C and cooling to 15 °C at a rate of 1 °C per 10 min. Atomic force microscopy (AFM) images (Figure 2b,c) revealed bright spots on the rDON sheets, corresponding to LL‐37 attachment. The height of these spots, ≈2 nm, was taller than the flat rDON (≈1 nm), confirming successful conjugation (Figure 2d). Accordingly, the loading efficiency of LL‐37 was calculated from AFM images by analyzing the number of LL‐37 on an rDON sheet. Quantitatively, the yield of rDON with 10 LL‐37 molecules reached 55.73% (Figure S4, Supporting Information). Also, as shown in the AGE data (Figure 2e), the CB‐rDONs@AMPs displayed slower mobility than CB‐rDON. Dynamic light scattering (DLS) further demonstrated a size increase from ≈81 nm for CB‐rDON to ≈110 nm for CB‐rDON@AMPs (Figure 2f). The attachment of the cationic peptide LL‐37 (+ 0.98 mV) also caused a shift in ζ potential from ≈ −3.0 mV for CB‐rDON to ≈ −1.8 mV for CB‐ rDON@AMPs (Figure 2g). Subsequently, we modified the rDON with varying amounts of LL‐37 (i.e., 2, 6, 10). As revealed in the AFM images (Figure 2h), the bright spots corresponding to the different amounts of attached peptides were clearly visible on the surface of each rDON sheet. We verified the antimicrobial ability of different amounts of LL‐37 against *E. coli* and *S. aureus* by agar plate experiments (Figure S5, Supporting Information). As shown in Figure 2i,j, the configuration with 10 antimicrobial peptides demonstrated the best antimicrobial effect. Therefore, we chose to modify each rDON with 10 LL‐37 molecules in subsequent experiments. In addition, most of the CB‐rDON@AMPs maintained their nanostructures over a 24 h period in the presence of bovine serum albumin (BSA) or fetal bovine serum (FBS) (Figure S6, Supporting Information), suggesting potential high stability in body fluid. These findings confirmed the successful assembly of CB‐rDON@AMPs nanosheets with the desired structural and functional characteristics.

### 2.3 Responsiveness of DNA Origami Nanoplatform In Vitro

**Figure 3:**
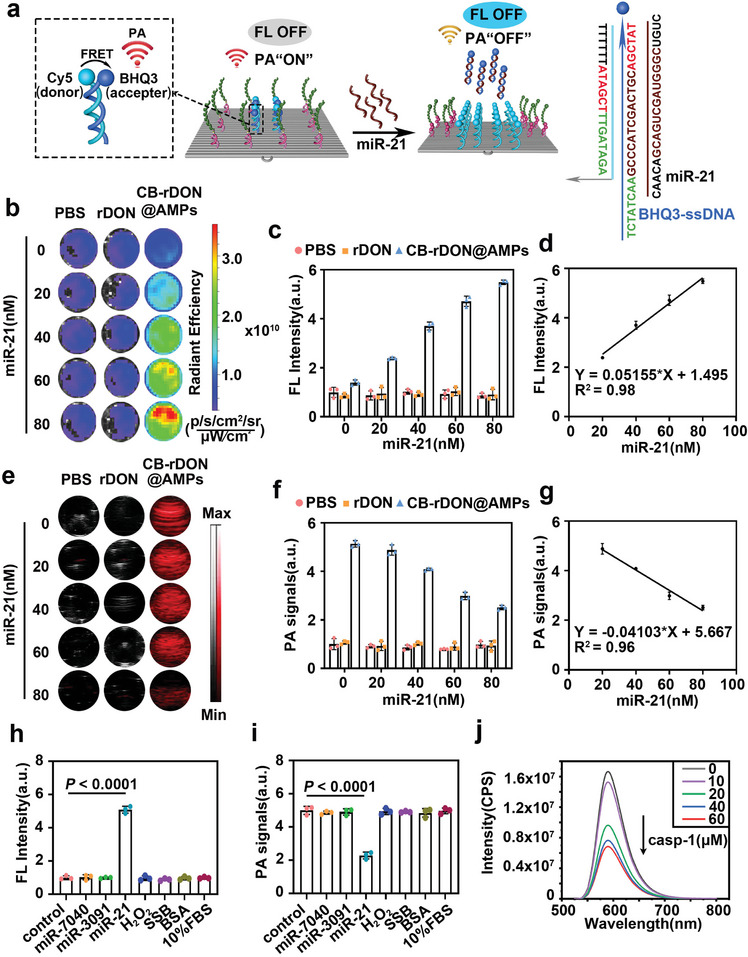
In vitro detection of miR‐21 using CB‐rDON@AMPs. a) Schematic representation of fluorescence activation and photoacoustic signal attenuation triggered by miR‐21 in CB‐rDON@AMPs. b–g) In vitro fluorescence (b) and photoacoustic (e) imaging of samples treated with PBS, rDON, and CB‐rDON@AMPs at various miR‐21 concentrations, with corresponding graphs showing normalized fluorescence (c) and photoacoustic (f) intensities. Graphs illustrating the relationship between miR‐21 concentration and normalized fluorescence (d) and photoacoustic signals (g). h,i) Normalized fluorescence (h) and photoacoustic intensity (i) of CB‐rDON@AMPs (10 nM, 200 µL) incubated with different molecules: miR‐7040 (80 nM), miR‐3091 (80 nM), miR‐21 (80 nM), H_2_O_2_ (125 µM), BSA (5 mM), SSB (5 mM), and 10% FBS. Data were presented as mean ± standard deviation (*n* = 3). Statistical analysis was performed using one‐way ANOVA. j) Emission spectra of CB‐rDON@AMPs‐FITC after incubation with varying concentrations of caspase‐1 (casp‐1).

The responsiveness of CB‐rDON@AMPs to miR‐21 and caspase‐1 was investigated in vitro. As illustrated in **Figure** **3**a, the presence of miR‐21 triggered a BHQ3‐ssDNA‐mediated strand displacement reaction on the CB‐rDON@AMPs, releasing BHQ3 from the DNA origami. This release separated BHQ3 from Cy5, restoring Cy5 fluorescence and reducing the photoacoustic signal. We evaluated the sensitivity of CB‐rDON@AMPs to increasing concentrations of miR‐21. As shown in Figure 3b,c, fluorescence intensity increased with rising miR‐21 levels, reaching over fivefold the baseline at 80 nM. A linear correlation was observed between the normalized fluorescence intensity of CB‐rDON@AMPs and miR‐21 concentration, with a detection limit of 1.50 nM (Figure 3d). Conversely, the photoacoustic (PA) signal exhibited a decreasing trend with increasing miR‐21 concentrations, achieving a detection limit of 5.67 nM (Figure 3e–g). We further confirmed the specificity of CB‐rDON@AMPs for miR‐21 by incubating the nanostructures with other microRNAs (miR‐7040 and miR‐3091) overexpressed in SA‐AKI, as well as H_2_O_2_, BSA, single‐stranded DNA‐binding protein (SSB), and 10% FBS. Fluorescence and photoacoustic imaging demonstrated high selectivity for miR‐21, with negligible responses to these other molecules (Figure 3h,i). These results confirmed the excellent specificity and selectivity of CB‐rDON@AMPs for miR‐21 detection. To further assess responsiveness to caspase‐1, LL‐37 was modified with the fluorescent dye FITC, creating CB‐rDON@AMPs‐FITC. As shown in Figure 3j, after incubation with different concentrations of caspase‐1, the emission spectra showed a gradual decrease in the fluorescence signal of FITC, indicating that LL‐37 was successfully cleaved from CB‐rDON@AMPs. In summary, the CB‐rDON@AMPs platform enabled sensitive and selective FL and PA detection of miR‐21, as well as responsiveness to caspase‐1 in vitro.

### 2.4 Predominant Accumulation of DNA Origami Nanoplatform in the Kidney and Fluorescence Diagnosis of SA‐AKI

**Figure 4:**
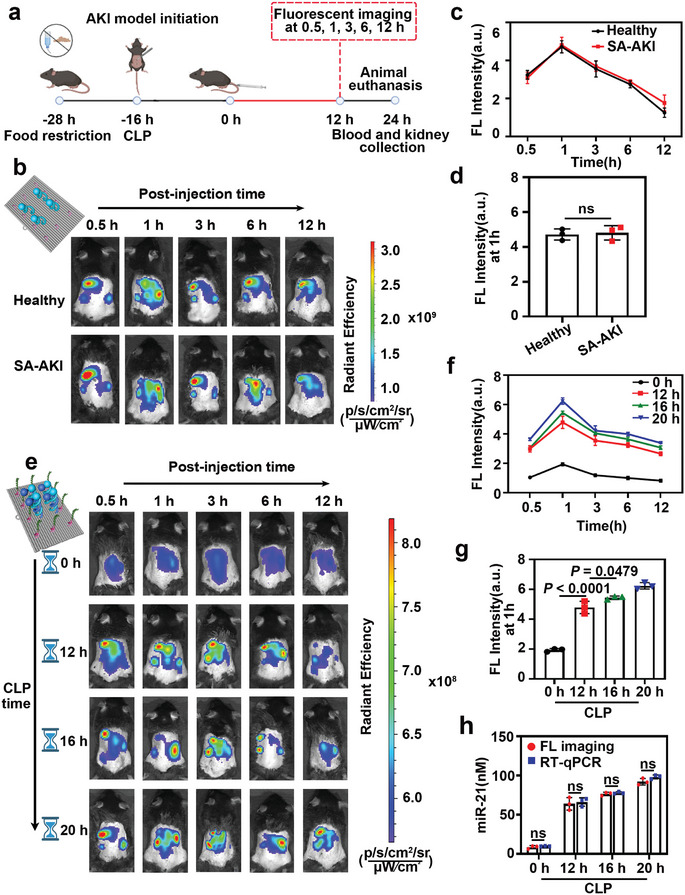
Biodistribution and fluorescence imaging of DNA origami nanoplatform in healthy and SA‐AKI mice. a) Schematic of the SA‐AKI mouse model and imaging timeline. b) Longitudinal fluorescence imaging of healthy and SA‐AKI mice at 0.5, 1, 3, 6, and 12 h after intravenous injection of C‐rDON@AMPs (20 nM, 200 µL; λ_ex_ = 620 nm, λ_em_ = 670 nm). Representative data from one of three independent experiments. c) Region of interest (ROI) analysis showing kidney accumulation at 0.5, 1, 3, 6, and 12 h post‐injection in both healthy and SA‐AKI mice. d) Statistical comparison of kidney fluorescence intensity at the 1 h mark between healthy and SA‐AKI mice. Data were presented as mean ± standard deviation (*n* = 3). Statistical analysis was performed using one‐way ANOVA. e) Longitudinal fluorescence imaging following CB‐rDON@AMPs administration (20 nM, 200 µL; λ_ex_ = 620 nm, λ_em_ = 670 nm) at the same time points. Data were from one of three independent experiments. f) ROI‐based quantification of kidney fluorescence intensity across time points in different groups of mice. g) Statistical comparison of kidney fluorescence intensity at 1 h among groups. Data were presented as mean ± standard deviation (*n* = 3). Statistical analysis was performed using one‐way ANOVA. h) Correlation analysis of miR‐21 levels measured by FL imaging and RT‐qPCR. Error bars denoted standard deviation from three independent measurements. Statistical significance was determined using two‐way ANOVA (ns denoted no significant difference).

We assessed the biodistribution of CB‐rDON@AMPs in healthy and SA‐AKI mice. Flow cytometry quantification of C‐rDON@AMPs (without BHQ3 modification) incubated with human embryonic kidney 293 (HEK‐293) cells for 4 h showed that the DNA origami nanoplatform could be transported into cells (Figure S7, Supporting Information). The blood half‐life (t_₁/₂_) of CB‐rDON@AMPs was ≈3.61 h post‐intravenous injection in healthy mice (Figure S8, Supporting Information). SA‐AKI model was established via cecal ligation and puncture (CLP). Elevated BUN and Cre (Figure S9, Supporting Information), rapid weight loss (Figure S10, Supporting Information), up‐regulation of miR‐21 (Figure S11, Supporting Information), and renal tubular epithelial cell edema and necrosis observed in hematoxylin and eosin (HE) sections of the kidney (Figure S12, Supporting Information), all of which demonstrated the success of the model. Following intravenous administration of C‐rDON@AMPs, we conducted fluorescence imaging at various time points (0.5, 1, 3, 6, and 12 h) in both healthy and SA‐AKI mice (λ_ex_ = 620 nm, λ_em_ = 670 nm) (**Figure** **4**a). Preferential renal accumulation was observed in both groups, as shown in Figure 4b. The fluorescence signal peaked at 1 h post‐injection (Figure 4c) in both groups, identifying this as the optimal diagnostic time point. However, no significant difference in fluorescence intensity was detected between healthy and SA‐AKI kidneys at this time (Figure 4d). The strong fluorescence in the urine collected at 24 h post injection (Figure S13, Supporting Information) indicated that the DNA origami platform could be metabolized out of the body through the kidney. To further investigate the potential for early diagnosis and severity assessment of SA‐AKI, mice with varying levels of AKI severity were generated by adjusting CLP durations (0, 12, 16, and 20 h). Fluorescence imaging after injection of CB‐rDON@AMPs showed higher fluorescence intensity in SA‐AKI kidneys, which allowed differentiation of severity compared to healthy controls (Figure 4e–g). Notably, the CLP‐12h group exhibited unchanged BUN and Cre levels (Figure S14, Supporting Information) but showed significant renal pathology (Figure S15, Supporting Information) and enhanced fluorescence. This indicated that CB‐rDON@AMPs can detect SA‐AKI before conventional clinical markers show abnormalities. In addition, as shown in Figure 4h, the concentration of miR‐21 measured by RT‐ qPCR (Table S6, Supporting Information) was comparable to that obtained through FL imaging, with no significant differences. To confirm that LL‐37 within CB‐rDON@AMPs could be cleaved in vivo, we synthesized CB‐rDON@AMPs‐FITC by conjugating LL‐37 with FITC, a molecule cleavable by caspase‐1. A mouse model of ischemia‐reperfusion (IRI)‐induced AKI was established by clamping the bilateral renal hilum for 30 min. Caspase‐1 concentrations in the kidneys of IRI‐AKI and SA‐AKI mice were then compared (Figure S16, Supporting Information). Following the injection of CB‐rDON@AMPs‐FITC, major organs were harvested at multiple time points (0.5, 1, 3, 6, and 12 h) for ex vivo imaging (λ_ex_ = 480 nm, λ_em_ = 520 nm). The FITC signal in IRI‐AKI kidneys was consistently higher than in SA‐AKI kidneys, indicating that elevated caspase‐1 levels facilitated the cleavage of LL‐37 from the nanoplatform (Figure S17, Supporting Information).

### 2.5 Photoacoustic Diagnosis of SA‐AKI Using a DNA Origami Nanoplatform

**Figure 5:**
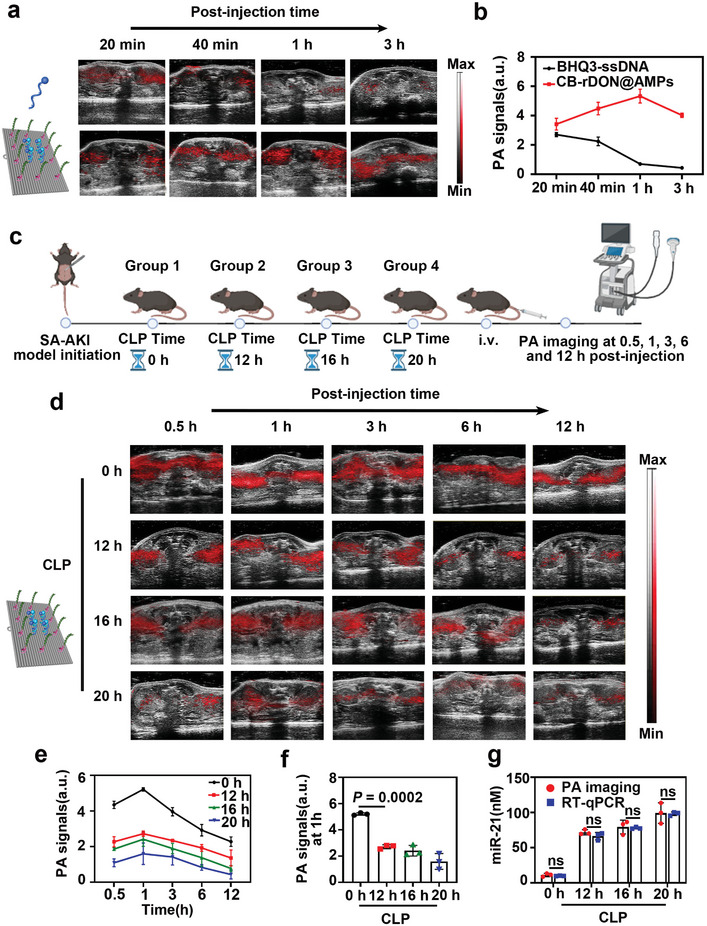
PA imaging for diagnosing SA‐AKI in mice. a) PA imaging of healthy mice following intravenous injection of BHQ3‐ssDNA (200 nM, 200 µL) and CB‐rDON@AMPs (20 nM, 200 µL). b) Region of interest (ROI) analysis of kidney PA signals recorded at 20 min, 40 min, 1 h, and 3 h post‐injection. Representative data were shown from one of three independent experiments. c) Schematic overview of healthy mice treated with different durations of CLP and the corresponding imaging timeline. d) Longitudinal PA imaging of mice exposed to varying CLP durations at 0.5, 1, 3, 6, and 12 h after CB‐rDON@AMPs injection. Data were shown from one of three independent experiments. e) ROI‐based quantification of kidney PA signals across time points for each group, from one of three independent experiments. f) Statistical comparison of kidney PA intensity at the 1 h mark among different groups. Data were presented as mean ± standard deviation (*n* = 3). Statistical analysis was performed using one‐way ANOVA. g), Correlation analysis of miR‐21 levels measured by PA imaging and RT‐qPCR. Data were presented as mean ± standard deviation (*n* = 3). Statistical significance was determined using two‐way ANOVA (ns denoted no significant difference).

We utilized the PA signal of BHQ3 to analyze metabolic differences between BHQ3‐ssDNA (≈40 nucleotides) and CB‐rDON@AMPs in mice. Healthy mice received intravenous injections via the tail vein of either BHQ3‐ssDNA or CB‐rDON@AMPs. PA imaging was performed at multiple time points using a 680 nm laser to monitor signal variations after injection. The PA images demonstrated a rapid decline in the PA signal of BHQ3‐ssDNA in the kidneys, with the signal becoming undetectable within 3 h. In contrast, the signal from CB‐rDON@AMPs progressively intensified, peaking 1 h post‐injection (**Figure** **5**a,b). These results suggested that the kidneys metabolize smaller, lower molecular weight DNA molecules faster than complex DNA nanostructures, highlighting the potential of PA signal dynamics for diagnosing SA‐AKI. As shown in Figure 5c, we tested this diagnostic capability by inducing SA‐AKI in mice using CLP of varying durations and performing PA imaging at 0.5, 1, 3, 6, and 12 h after injection of CB‐rDON@AMPs. As shown in Figure 5d, the most pronounced PA signal changes occurred in the kidneys 1 h post‐injection, consistent with fluorescence imaging trends. Quantitative analysis of PA signals at the 1 h mark revealed significant differences between mice with SA‐AKI and those without kidney injury, demonstrating the diagnostic potential of PA imaging (Figure 5e,f). Importantly, PA imaging was also effective in detecting early‐stage AKI, as confirmed by the results at 12h post‐CLP induction. Furthermore, we estimated miR‐21 concentrations based on PA signal intensity 1 h after injection by applying the linear relationship shown in Figure 3g. These estimates aligned closely with RT‐qPCR results, confirming the accuracy of PA imaging for quantifying miR‐21 levels in SA‐AKI (Figure 5g). These findings underscored the reliability of PA imaging for early diagnosis of SA‐AKI.

### 2.6 Antimicrobial and Antioxidant Efficacy of DNA Origami Nanoplatform In Vitro

Next, we evaluated the bactericidal capacity of CB‐rDON@AMPs in vitro. As shown in Figure S18 (Supporting Information), CB‐rDON@AMPs exhibited bactericidal activity comparable to LL‐37 alone, indicating that the antimicrobial properties of LL‐37 remained intact when conjugated to the rDON structure. We further explored the therapeutic potential of CB‐rDON@AMPs by developing a cellular inflammation model. HEK‐293 cells were treated with lipopolysaccharide (LPS) to mimic the inflammatory conditions characteristic of SA‐AKI. DNA origami structures, known for their significant antioxidant activity, were tested for their ability to scavenge ROS. ABTS radical scavenging experiments confirmed that the dye and peptide modifications on CB‐rDON@AMPs did not impair their ROS‐neutralizing efficiency (Figure S19, Supporting Information). We assessed the cytoprotective effect of CB‐rDON@AMPs on LPS‐treated HEK‐293 cells through MTT assays. The results indicated a significant improvement in cell viability following CB‐rDON@AMPs treatment, outperforming rDON or LL‐37 when used independently (Figure S20, Supporting Information). Confocal microscopy and live/dead cells fluorescence staining further corroborated these findings, demonstrating enhanced cellular survival in treated samples (Figure S21, Supporting Information). Collectively, these results suggested that CB‐rDON@AMPs offer superior therapeutic efficacy by combining antimicrobial action and ROS scavenging capabilities, positioning them as a promising intervention for SA‐AKI.

### 2.7 Therapeutic Efficacy of DNA Origami Nanoplatform in SA‐AKI Mouse Models

**Figure 6:**
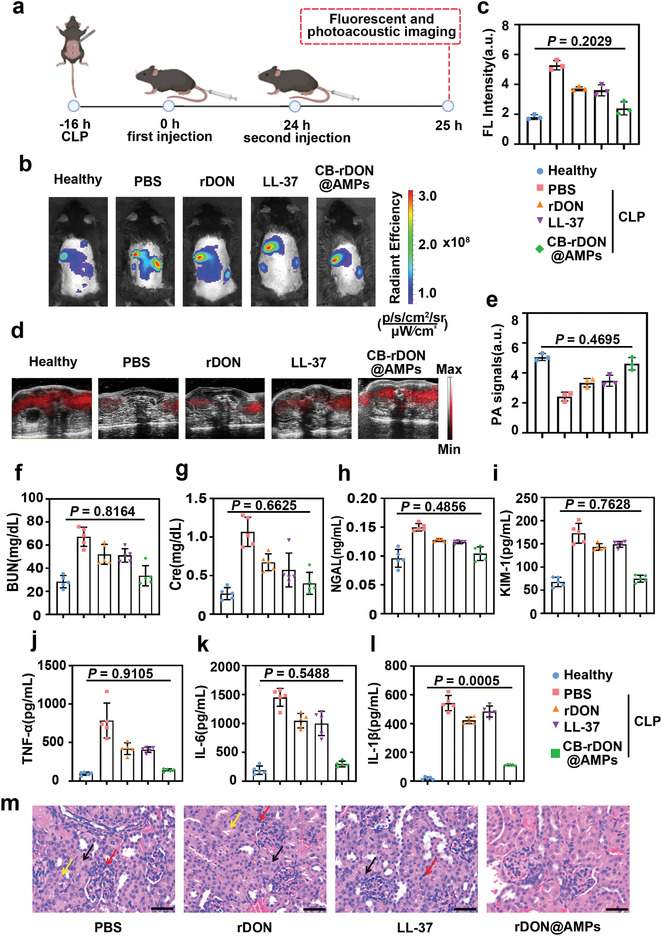
Therapeutic evaluation of dual‐responsive origami nanoplatform for SA‐AKI treatment. a) Schematic representation of the CB‐rDON@AMPs‐based therapeutic strategy for SA‐AKI mice. b) Fluorescence imaging of SA‐AKI mice treated with PBS, LL‐37 (200 nM, 200 µL), rDON (20 nM, 200 µL), or CB‐rDON@AMPs (20 nM, 200 µL). c) Quantitative comparison of FL intensity in the kidneys across treatment groups at 1 h post‐injection. d) PA imaging of SA‐AKI mice following the same treatments as in panel b. e) Statistical analysis of PA signal intensities in the kidneys for different groups at 1 h post‐injection. f–l), Measurement of BUN (f), Cre (g), NGAL (h), KIM‐1 (i), TNF‐α (j), IL‐6 (k), and IL‐1β (l) levels in SA‐AKI mice 24 h after treatment. Data were presented as mean ± standard deviation (*n* = 5). Statistical comparisons were performed using one‐way ANOVA. m, Representative H&E‐stained micrographs of kidney tissue from different groups (*n* = 5). Yellow arrows indicate tubular epithelial cell swelling, black arrows denote necrosis of tubular epithelial cells, and red arrows highlight inflammatory cell infiltration (Scale bars = 40 µm).

To validate the therapeutic efficacy in vivo, we conducted therapeutic assessments on four groups of SA‐AKI mice: 1) PBS buffer, 2) LL‐37 (200 nM, 200 µL), 3) rDON (20 nM, 200 µL), and 4) CB‐rDON@AMPs (20 nM, 200 µL). Treatments were administered intravenously 16 h post‐CLP, with a second dose of CB‐rDON@AMPs given 24 h later. 1 h after the second injection, fluorescence and PA imaging were performed to monitor kidney function and treatment efficacy (**Figure** **6**a). Both imaging modalities revealed that CB‐rDON@AMPs outperformed other treatments. Fluorescence signals in the CB‐rDON@AMPs‐treated group were reduced by ≈55% relative to the untreated group (Figure 6b,c). Similarly, PA imaging demonstrated a 47% increase in signal intensity in the treated group compared to controls (Figure 6d,e), confirming the superior effectiveness of CB‐rDON@AMPs. After imaging, blood and kidney tissues were collected from all groups to assess AKI biomarkers and perform histological analysis. CB‐rDON@AMPs significantly improved kidney function, reducing BUN levels to 37.5 mg dL^−1^ and Cre to 0.5 mg dL^−1^, approximating healthy control levels (Figure 6f,g). Additionally, early kidney injury markers kidney injury molecule‐1 (KIM‐1) and neutrophil gelatinase‐associated lipocalin (NGAL) were markedly reduced (Figure 6h,i), further demonstrating therapeutic efficacy.

To evaluate the anti‐inflammatory effect of CB‐rDON@AMPs, we quantified key cytokines IL‐6, IL‐1β, and TNF‐α using enzyme linked immunosorbent assay (ELISA). CB‐rDON@AMPs significantly suppressed inflammatory cytokines in the kidney (Figure 6j–l), highlighting the synergistic effects of LL‐37 and rDON. Histological examination using HE staining revealed near‐complete recovery of renal tubules with minimal inflammatory infiltration in the CB‐rDON@AMPs group. In contrast, the PBS, LL‐37, and rDON groups exhibited severe kidney damage, including tubular dilation, brush border loss, and persistent inflammation (Figure 6m), underscoring the limited efficacy of LL‐37 or rDON alone. Survival analysis further corroborated these findings. Over a seven‐day observation period, the CB‐rDON@AMPs group showed a survival rate of 90%, significantly higher than the 10% survival observed in the PBS group (Figure S22, Supporting Information). These results demonstrated the potent therapeutic potential of CB‐rDON@AMPs in mitigating SA‐AKI.

### 2.8 Safety of DNA Origami Nanoplatform

To evaluate the safety profile of the DNA origami platform, we first performed methyl thiazolyl tetrazolium (MTT) assays to assess cytotoxicity. As shown in Figure S23 (Supporting Information), cell viability of HEK‐293 cells exceeded 98.7% after 24 h of incubation with 20 nm CB‐rDON@AMPs. These findings demonstrated the low cytotoxicity of CB‐rDON@AMPs in vitro. We further evaluated liver and kidney function in healthy mice treated with CB‐rDON@AMPs using blood biochemistry and routine hematology analyses. The results, presented in Figure S24 (Supporting Information), showed that all measured parameters remained within normal ranges. This indicated the negligible in vivo toxicity of CB‐rDON@AMPs. Additionally, H&E staining of major organs from treated mice (Figure S25, Supporting Information) revealed no significant histopathological changes in any biopsy samples, underscoring the excellent biocompatibility of CB‐rDON@AMPs.

## 3 Conclusion

Here, we designed a novel DNA origami nanoplatform, CB‐rDON@AMPs, to enable both precise diagnosis and effective treatment of SA‐AKI. This multifunctional platform exploited the kidney‐targeting properties of rDON, facilitating rapid accumulation in renal tissue within 1 h of intravenous administration. Once inside the kidney, the nanoplatform interacted with the overexpressed miR‐21, a biomarker prevalent in AKI‐affected tissue. This interaction triggered BHQ3 to dissociate from Cy5 on the DNA origami nanostructure, resulting in fluorescence activation and PA signal attenuation. This mechanism offered early SA‐AKI detection, outperforming traditional markers such as BUN and Cre, which often detected kidney damage only at later stages. In addition to its diagnostic capacity, CB‐rDON@AMPs provided therapeutic benefits. The AMPs on the nanoplatform acted synergistically with rDON's ROS scavenging activity, mitigating oxidative stress and inflammation. These combined actions improved survival outcomes, significantly extending the lifespan of treated mice. The use of DNA origami nanostructures as carriers introduces a new paradigm for multifunctional disease management, merging diagnostic and therapeutic modalities into a single platform.

## Conflict of Interest

The authors declare no conflict of interest.

## Supporting information



Supporting Information

## Data Availability

The data that support the findings of this study are available from the corresponding author upon reasonable request.
