# Correction to “A Dual‐Response DNA Origami Platform for Imaging and Treatment of Sepsis‐Associated Acute Kidney Injury”

**DOI:** 10.1002/advs.202510994

**Published:** 2025-07-04

Yingying Zhao, Yadan Zhao, Yufan Ling, Zhiming Chen, Xiaofeng Wu, Xing Lu^*^, Yao He^*^, Houyu Wang^*^ and Fenglin Dong^*^

*Adv. Sci*. **2025**;12(16): e2416330, DOI: 10.1002/advs.202416330

Following our careful check of all raw data in our published work, we discovered that one figure panel was used in error in Figure 2b. The authors identified the mistake, and the corrected Figure 2b is as follows:


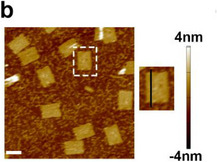


Figure 2. b) Representative atomic force microscopy (AFM) images of CB‐rDON. Scale bars = 50 nm.

We apologize for the error. The correction does not affect the conclusion drawn from the paper.

