# Supplementary material for: A Dual‐Response DNA Origami Platform for Imaging and Treatment of Sepsis‐Associated Acute Kidney Injury
Source: Adv Sci (Weinh). 2025 Feb 28;12(16):2416330. doi: 10.1002/advs.202416330 (PMC12021062; doi:10.1002/advs.202416330)
Supplement: Supplementary file 1 — Supporting Information [file ADVS-12-2416330-s001.docx]

Supporting Information
©Wiley-VCH 2021
69451 Weinheim, Germany

**A Dual-Response DNA Origami Platform for Imaging and Treatment of Sepsis-Associated Acute Kidney Injury**

Yingying Zhao^1^, Yadan Zhao^2^, Yufan Ling^3^, Zhiming Chen^4^, Xiaofeng Wu^1^, Xing Lu^2,^* , Yao He^2,5,6,^*, Houyu Wang^2,^* & Fenglin Dong^1,^*

**Abstract:** Current diagnostics for sepsis-associated acute kidney injury (SA-AKI) detect kidney damage only at advanced stages, limiting opportunities for timely intervention. We developed a DNA origami-based nanoplatform for the early diagnosis and treatment of SA-AKI. Modified with a fluorophore (Cy5) and quencher (BHQ3), the DNA origami remains non-fluorescent under normal conditions. During SA-AKI, elevated microRNA-21 triggers a strand displacement reaction that restores the fluorescence signal, enabling real-time detection. Additionally, the photoacoustic changes of BHQ3, driven by different excretion rates of the nanostructure and released DNA strands, enable dual-mode imaging, enhancing diagnostic accuracy. Therapeutically, DNA origami scavenges reactive oxygen species and, when conjugated with the antimicrobial peptide LL-37, exhibits bactericidal effects. This combination boosts survival rates by 80% in SA-AKI models. This dual-response nanoplatform integrates precise imaging and targeted therapy, offering a powerful strategy for SA-AKI management and advancing applications of DNA origami in precision nanomedicine.

**DOI: 10.1002/anie.2021XXXXX**

Experimental Procedures

**Materials:** All DNA staples, ranging between 20 and 60 bases (100 μM), were obtained from Sangon Biotech and were directly utilized in the self-assembly process without further purification. The M13mp18 single-stranded DNA scaffold (m13 ssDNA) was procured from New England Biolabs (catalog no.: N4040S). Antimicrobial peptide LL-37 was sourced from Aladdin (catalog no.: 154947-66-7), while the peptide substrate Tyr-Val-Ala-Asp (YVAD) came from QYAOBIO. NHS-FITC and enzyme-linked immunosorbent assay (ELISA) kits for TNF-α, IL-6, IL-1β, NGAL, and KIM-1 were supplied by Thermo Fisher Scientific. Additional ELISA kits for NGAL and KIM-1 were obtained from LunChangShuo Biotech. Hoechst33342 and ROS assay kits, along with Calcein/PI cell viability kits, were acquired from Beyotime.

**Animals and housing conditions:** Adult male C57BL/6 mice (weighing 25–30 g) were supplied by Changzhou Cavens Laboratory Animal Co., Ltd. The animals were housed in pairs, maintained at 20–22 °C with 40–60 % humidity under a 12-hour light/dark cycle. They had unrestricted access to standard chow and sterile water. Animal health was monitored daily, with no animals displaying severe illness or succumbing to experimental procedures. All the animal experiments were conducted in accordance with the animal experiment protocols approved by the Soochow University Laboratory Animal Center (approval number: syxk (su) 2021--0073).

**Synthesis and characterization of CB-rDONs.** CB-rDONs were synthesized by annealing the viral genome M13mp18 (M13 ssDNA, 7,249 nucleotides) with complementary short staple and functional strands in 1× TAE-Mg²⁺ buffer (40 mM Tris, 20 mM acetic acid, pH 8.0, 2 mM EDTA, 12.5 mM Mg(CH₃COOH)₂). A molar ratio of 8:1 (short strands to M13) was maintained. The annealing process involved gradual cooling from 95 °C to 25 °C (temperature gradient in Table S5). Excess short strands were eliminated using Amicon Ultra 100 kD filters (Millipore), followed by three washes with TAE-Mg²⁺ buffer. Samples were analyzed using 1% agarose gel electrophoresis (AGE), atomic force microscopy (AFM), dynamic light scattering (DLS), and zeta potential measurements. AGE was conducted in TAE-Mg²⁺ buffer at 90 V for 85 min, with stained bands imaged on a chemiluminescence system.

**Preparation of CB-rDON@AMPs conjugates.** The peptide linker (YVAD; 0.25 mg/mL) was modified by introducing NHS at its tyrosine terminus and maleimide at the aspartic acid end. This modified linker was combined with LL-37 (0.5 mg/mL) at a 50:1 molar ratio. Covalent bonding between the linker and LL-37 was achieved through condensation between the NHS group and the amino group of the peptide, with the mixture incubated at 25 °C for one hour. The unreacted linker was removed via centrifugation at 7,000 × g for 30 minutes, followed by collection of the remaining solution. Thiolated ssDNA was added in 15-fold excess and incubated overnight at 4 °C to facilitate binding. The final conjugates were purified using 3 kD centrifugal filters. These conjugates were further mixed in a 10-fold excess with CB-rDON containing LL-37 capture strands, using 1× TAE-Mg²⁺buffer. The solution was heated to 37 °C and gradually cooled to 15 °C in 10 °C intervals to promote annealing. Excess conjugates were removed with 100 kD centrifugal filters.

**Establishment of SA-AKI mice models.** The SA-AKI models were created using cecal ligation and puncture (CLP) with a 22-gauge needle, followed by a subcutaneous injection of saline (1 mL per 25 g of body weight). Control mice underwent surgery without the puncture step. Successful establishment of the model was confirmed through renal histology (H&E staining), measurements of blood urea nitrogen (BUN), serum creatinine (Cre) levels, weight monitoring, and miR-21 expression.

**In vivo FL and PA imaging.** For FL imaging, 20 nM CB-rDON@AMPs (200 µL) was intravenously administered to C57BL/6 mice with SA-AKI. The imaging was performed using the IVIS Lumina III optical system at intervals of 0.5, 1, 3, 6, and 12 hours post-injection. Fluorescence intensity over time was calculated from the region of interest (ROI) to evaluate renal signal dynamics. PA imaging was conducted at 680 nm with 40 dB gain using a 21 MHz transducer (FUJIFILM Visual Sonics). The transducer-target distance was maintained at 5–7 mm, with a focal depth ranging from 5–12 mm.

**Quantification of renal miR-21 expression.** Real-time PCR was carried out on a Bio-Rad CFX96 system with 2× miRNA qPCR master mix (Sangon Biotech). The reaction protocol included an initial denaturation at 95 °C for 30 seconds, followed by 40 cycles of 95 °C denaturation for 5 seconds and amplification at 60 °C for 30 seconds. Calibration curves were established using serial dilutions of miR-21 templates (10⁹ to 10¹ copies). Primer sequences are detailed in Table S6.

**Blood biochemical analysis.** Mice were euthanized 24 hours post-injection, and blood samples were collected into EP tubes. After standing for 1 hour, the samples were centrifuged at 3,000 × g for 15 minutes at 4 °C. Levels of BUN, Cre, NGAL, KIM-1, TNF-α, IL-6, and IL-1β were measured using commercial assay kits.

**Tissue analysis and histological examination.** Kidney tissues were harvested 24 hours after SA-AKI induction. The tissues were fixed in 4% paraformaldehyde, embedded in paraffin, sectioned, and stained with hematoxylin and eosin (H&E). The sections were prepared by Suzhou Koch Biotechnology Co., Ltd.

**Cellular ROS scavenging assay.** HEK-293 cells were cultured in DMEM supplemented with 10% FBS and 1% penicillin-streptomycin at 37 °C and 5% CO₂. Cells (20,000 per well) were seeded in 96-well plates and incubated for 24 hours. After LPS stimulation (10 µg/mL) for 4 hours, PBS, rDON (20 nM), LL-37 (200 nM), and CB-rDON@AMPs (20 nM) were separately added and incubated for 30 minutes. The intracellular ROS levels were quantified using a DCFH-DA assay (Beyotime, S0033S). Laser scanning confocal microscopy (LSCM) was used to image the ROS, with excitation/emission wavelengths of 488/525 nm.

**Cell viability and therapeutic evaluation.** HEK-293 cells were cultured and treated similarly for the therapeutic evaluation. Following LPS stimulation, cells were incubated with PBS, rDON, LL-37, or CB-rDON@AMPs for 30 minutes. Cell viability was assessed using the Calcein/PI Cell Viability Assay (Beyotime, C2015S). Calcein-stained live cells emitted green fluorescence (λ_ex_ /λ_em_ = 494/517 nm), while PI-stained dead cells fluoresced red (λ_ex_ /λ_em_ = 535/617 nm). Imaging was performed using LSCM.

**Statistical analysis.** Data were analyzed using GraphPad Prism 7.0. A two-tailed paired one-way ANOVA was used to determine statistical significance. Results are presented as mean ± standard deviation from three independent experiments, with error bars indicating variability.

**Life Science Reporting Summary.** Further information on the experimental design is available in the Life Science Reporting Summary.

**Data availability.** The data that support the findings of this study are available within the paper and its supplementary information. Source data are provided with this paper.

Results and Discussion


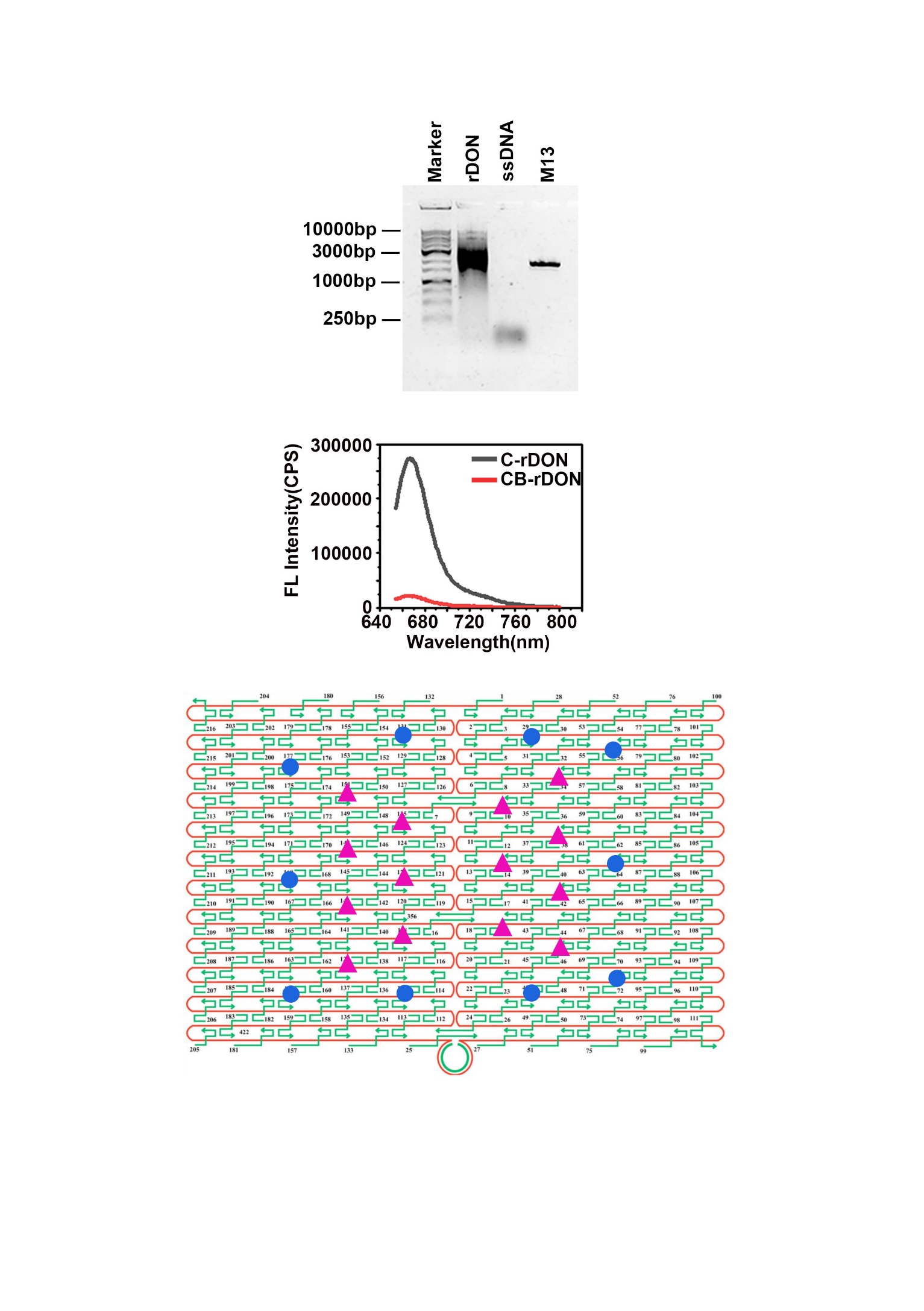


**Figure S1.** The DNA origami design includes modification sites for Cy5 and BHQ3, as well as functional strands for LL-37 capture. Fourteen sites for modified Cy5 strands (positions 10, 14, 19, 34, 38, 42, 46, 118, 122, 125, 139, 143, 147, and 151, marked in red) are extended to capture BHQ3-ssDNA. Additionally, ten LL-37 capture strands (positions 29, 47, 56, 64, 72, 115, 131, 161, 169, and 177, marked in blue) are extended at the 5’ end with ten binding sites for capturing LL-37-ssDNA.

**
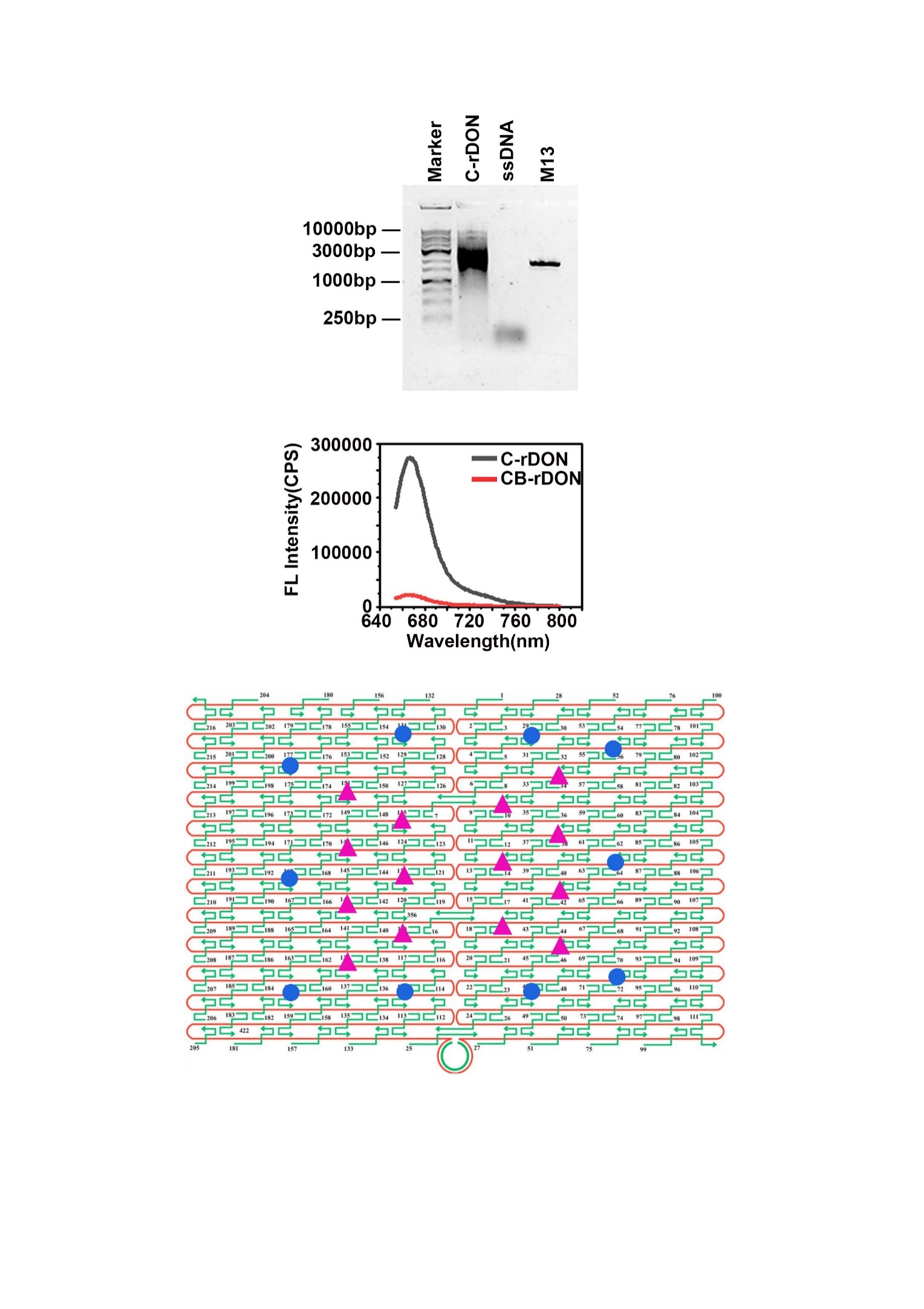
**

**Figure S2.** AGE(1%) data of C-rDON, ssDNA, and M13mp18 genomic DNA strands (M13). The larger C-rDON exhibited slower mobility compared to M13. All imaging experiments were repeated three times, yielding similar results.


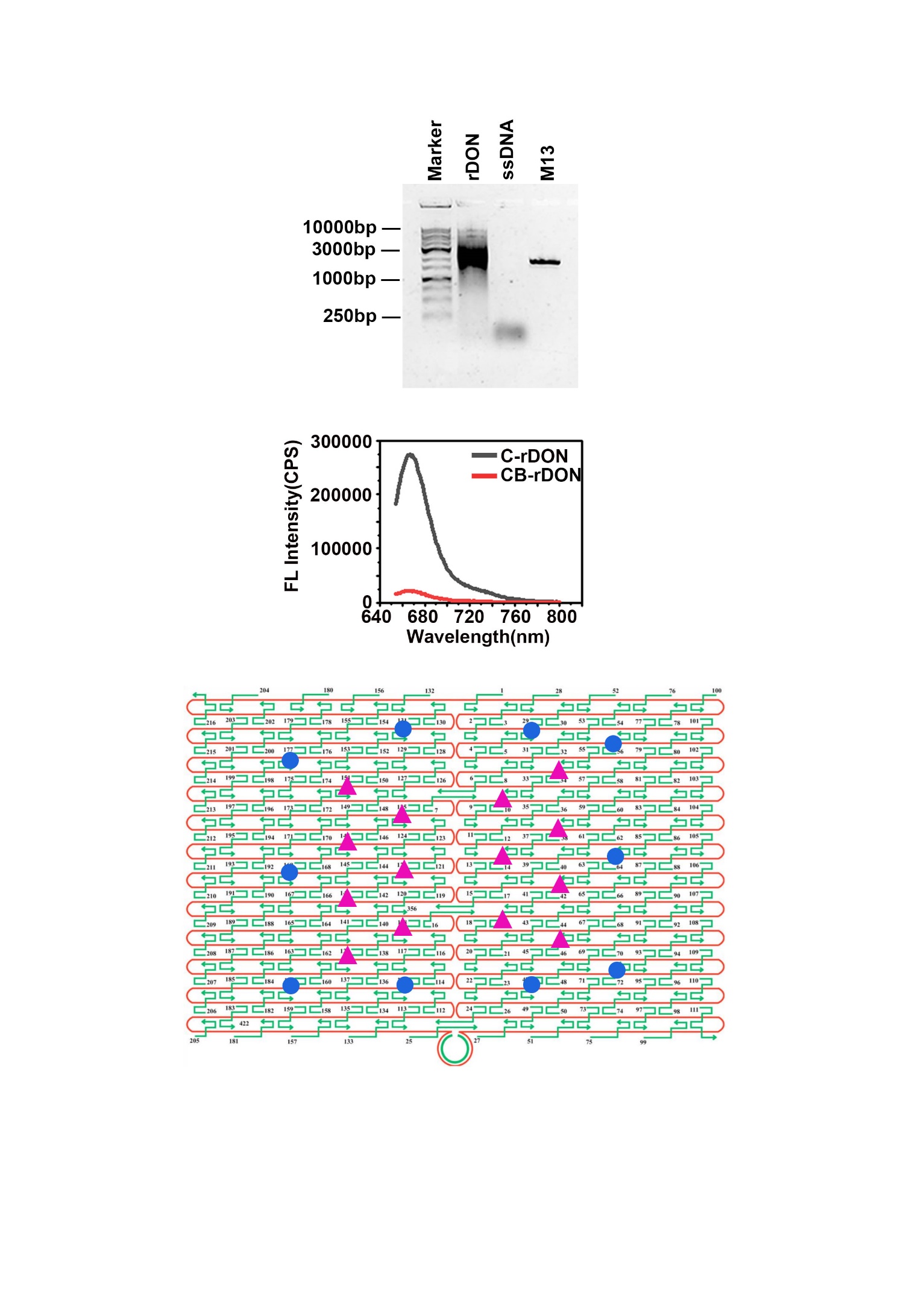


**Figure S3.**  Emission spectra of C-rDON and CB-rDON. The fluorescence of Cy5 was quenched by BHQ3, indicating the successful synthesis of CB-rDON (λ_ex_ = 650 nm, λ_em_ = 670 nm).


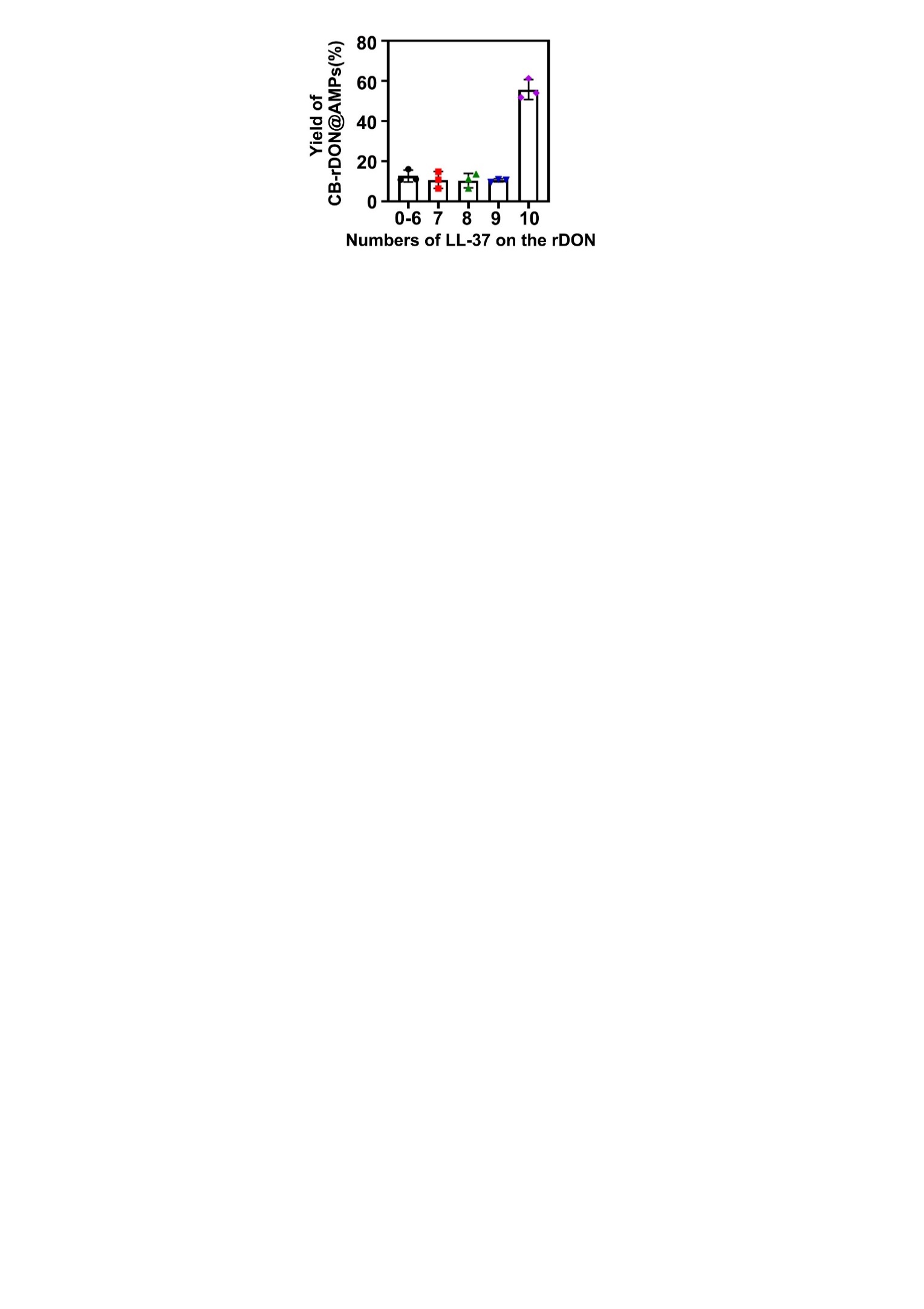


**Figure S4.** Yield of CB-rDON@AMPs. Yield of LL-37 molecules loaded onto rDON in different amounts. Error bars represent the standard deviation based on three independent measurements.


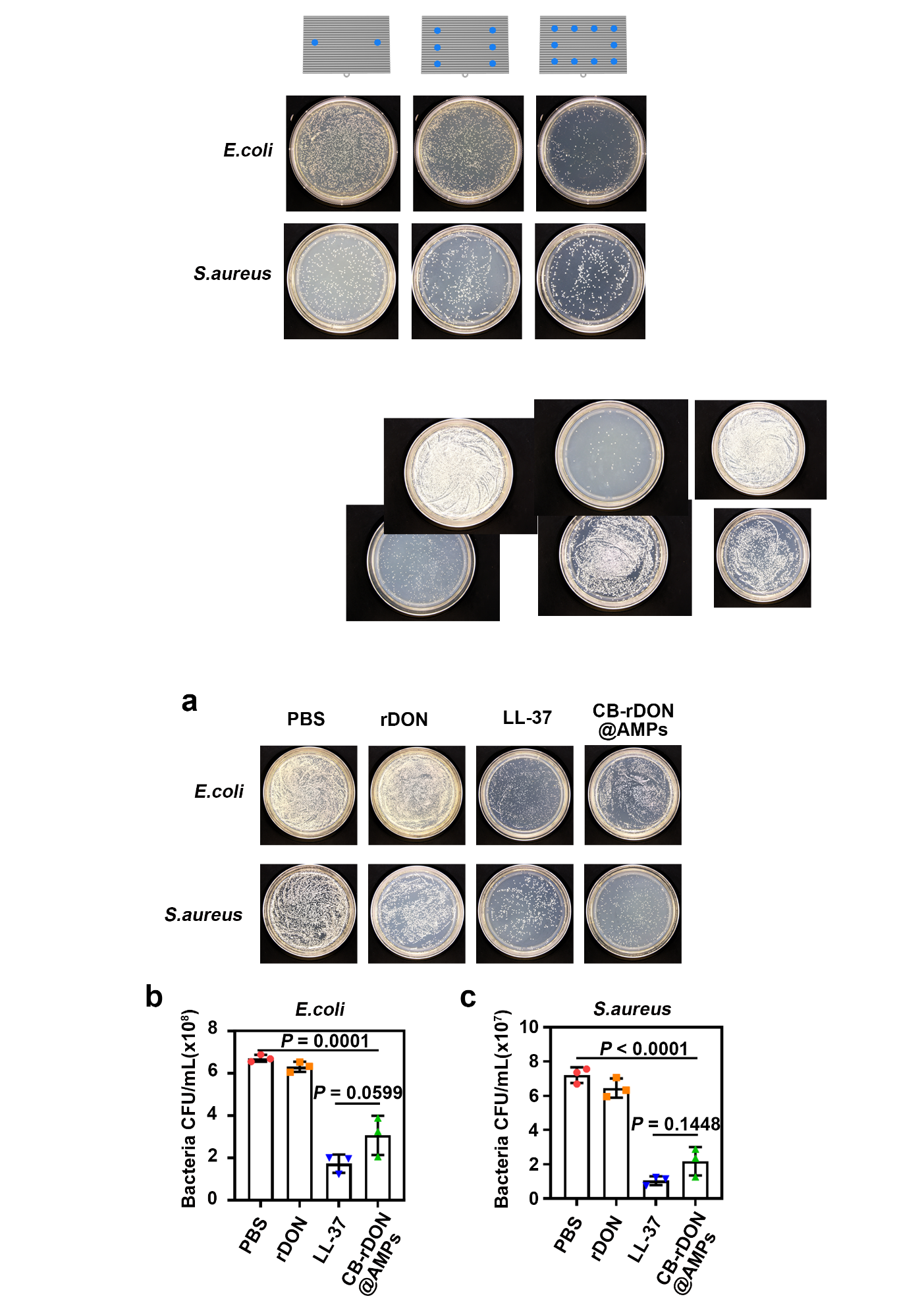


**Figure S5.** Photographs of agar plates showing *E. coli* and *S. aureus* treated with CB-rDON@AMPs conjugated with 2, 6, and 10 LL-37 molecules, respectively. The CB-rDON@AMPs containing 10 antimicrobial peptides exhibited the most potent antimicrobial effect. All imaging experiments were performed in triplicate, and consistent results were obtained.

**
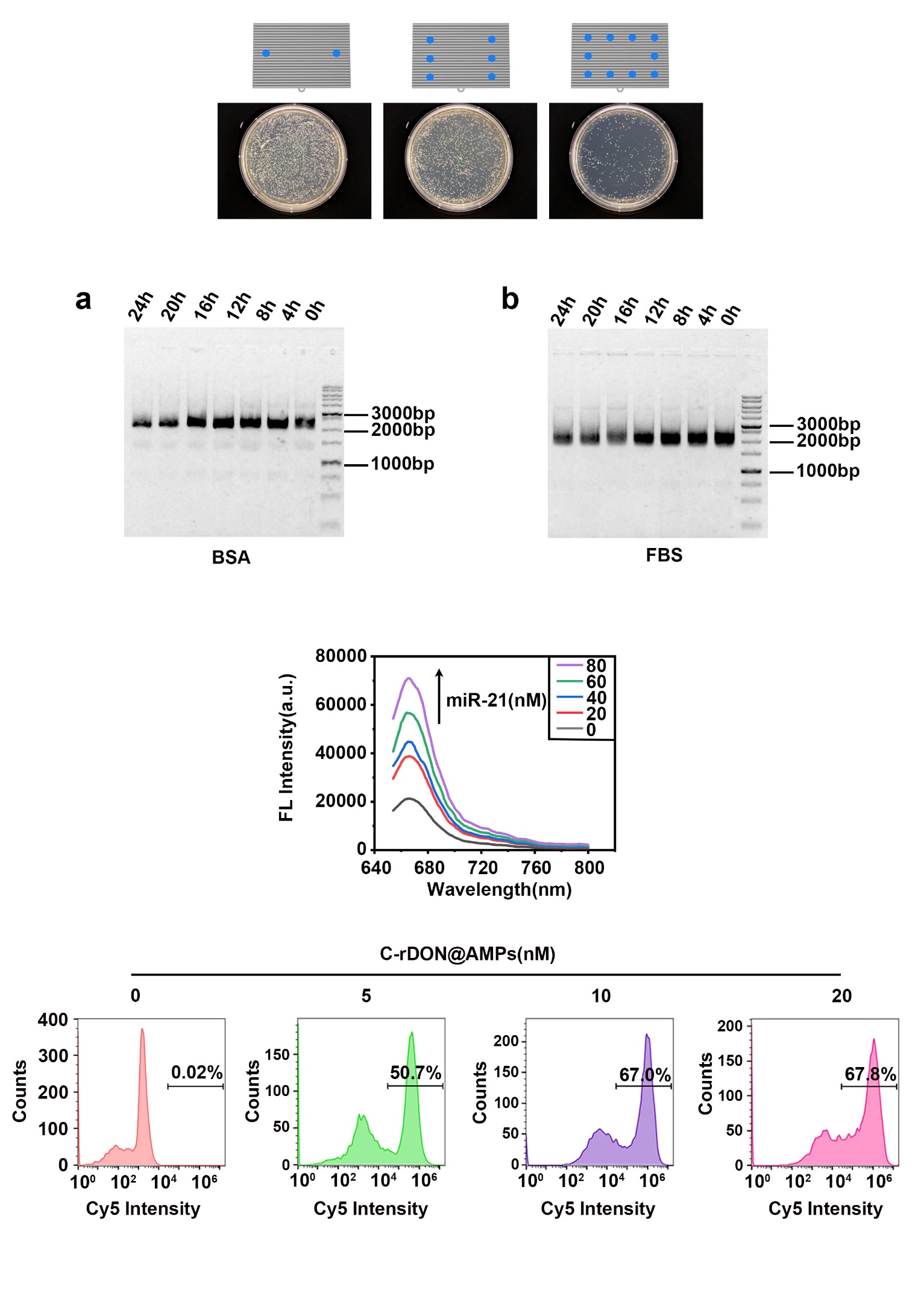
**

**Figure S6.** The good stability of CB-rDON@AMPs in bovine serum albumin (BSA) or fetal bovine serum (FBS). (a) Agarose gel electrophoresis of CB-rDON@AMPs (5 nM) incubated in 2% BSA for varying lengths of time. (b) Agarose gel electrophoresis of CB-rDON@AMPs (5 nM) incubated in 10% FBS for varying lengths of time. All imaging experiments were repeated three times, yielding similar results.


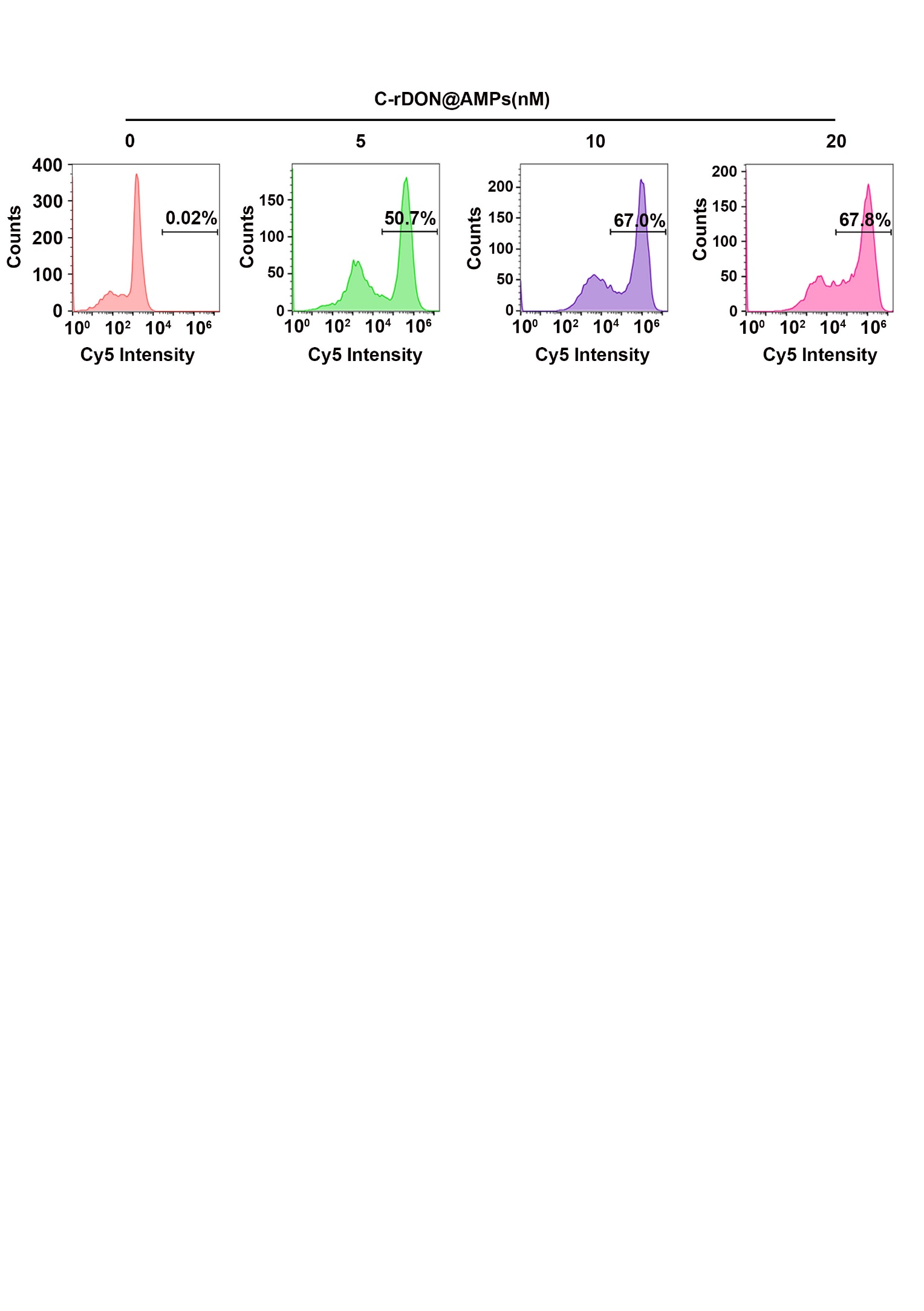


**Figure S7.** Flow cytometry analysis of cell internalization. Quantification of C-rDON@AMPs uptake by HEK-293 cells after 4 hours of incubation was performed using flow cytometry. Results from one of three independent experiments are presented.


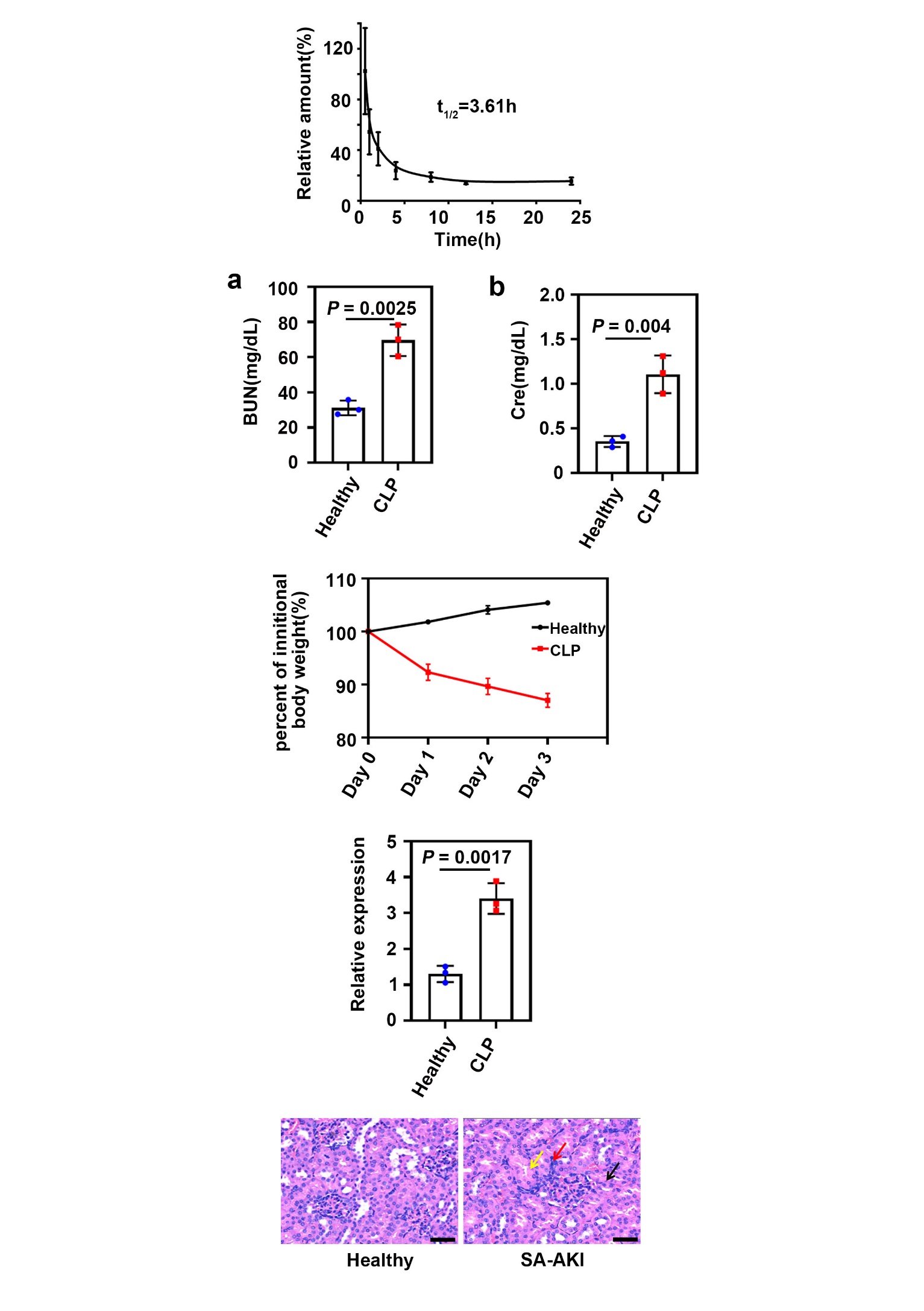


**Figure S8.** The blood half-life (t₁/₂) of CB-rDON@AMPs. Relative amount of CB-rDON@AMPs remained in the blood at 0.5, 1, 2, 4, 8, 12 and 24 h after *i.v.* administration into healthy mice. Error bars represent the standard deviation obtained from three independent measurements.

**
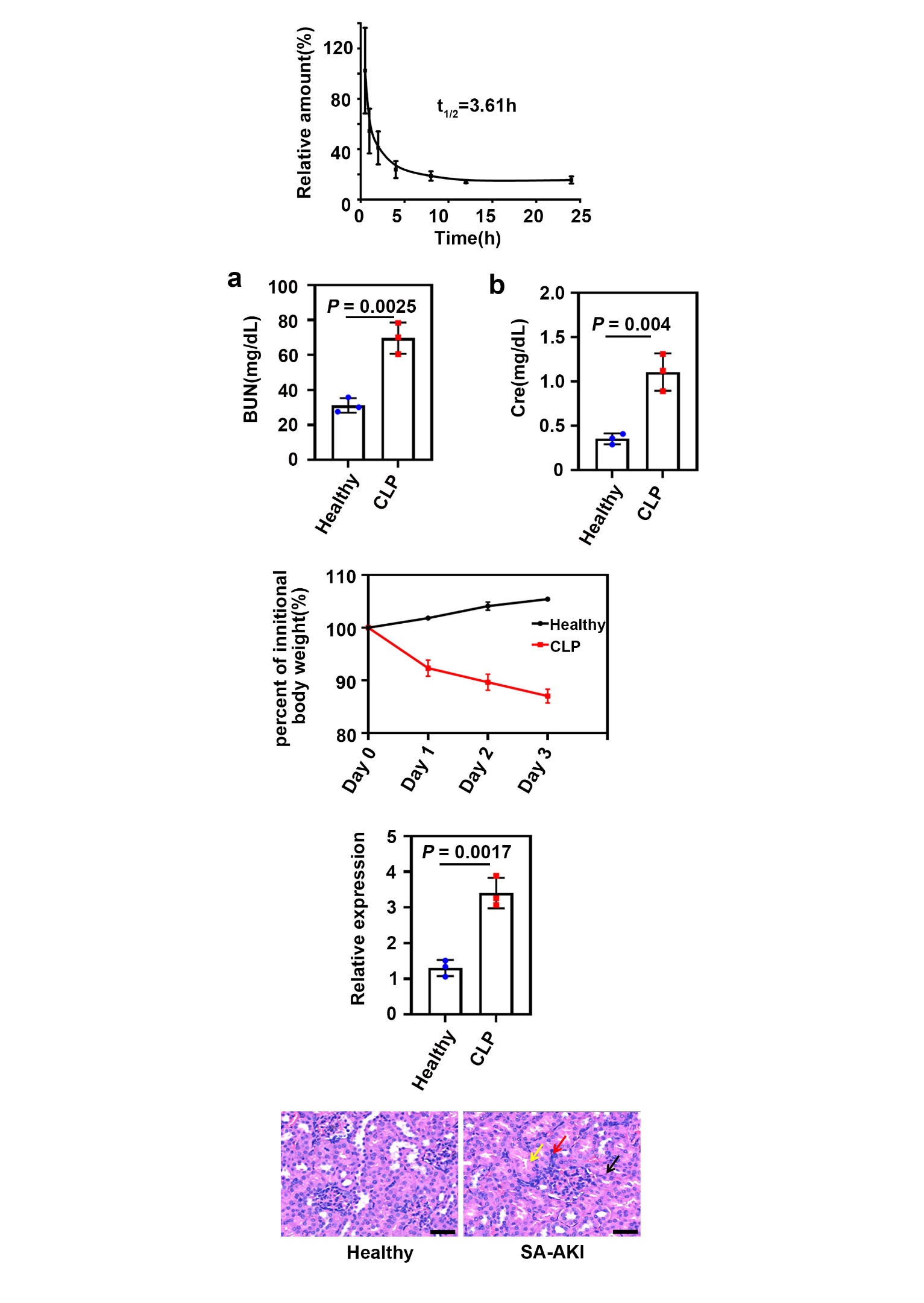
**

**Figure S9.** Serum biochemical parameters of healthy and SA-AKI mice. (a) Blood urea nitrogen(BUN) concentration. (b) Serum creatinine(Cre) concentration. Error bars represent the standard deviation obtained from three independent measurements. Statistical analysis was performed using one-way ANOVA.


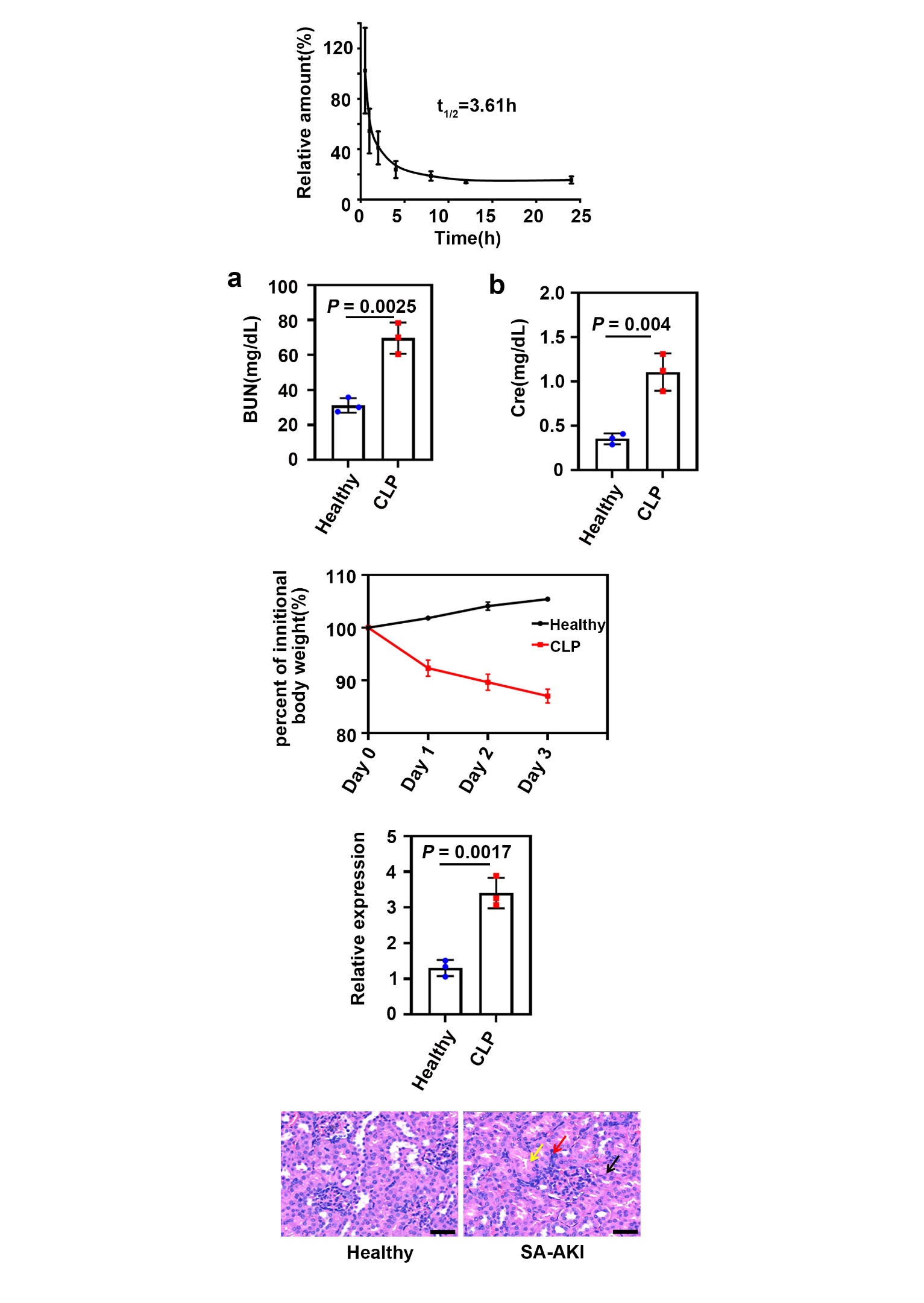


**Figure S10.** Changes of mouse body weight. The body weight changes of healthy and SA-AKI mice in 3 days. Error bars represent the standard deviation obtained from three independent measurements.


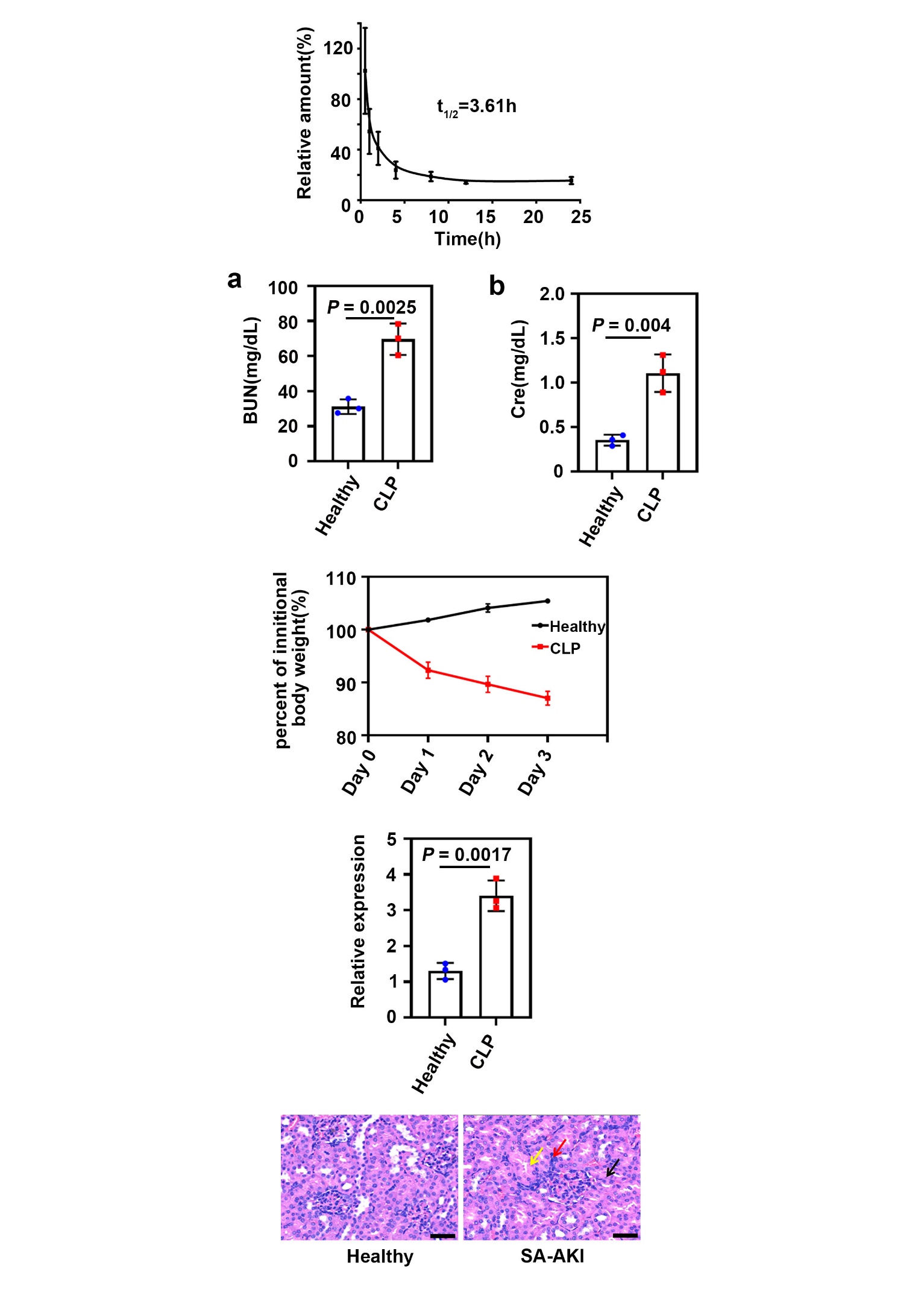


**Figure S11.** Relative expression of miR-21. RT-qPCR of renal miR-21 of healthy and SA-AKI mice post different treatments as indicated. Error bars represent the standard deviation obtained from three independent measurements. Statistical analysis was performed using one-way ANOVA.


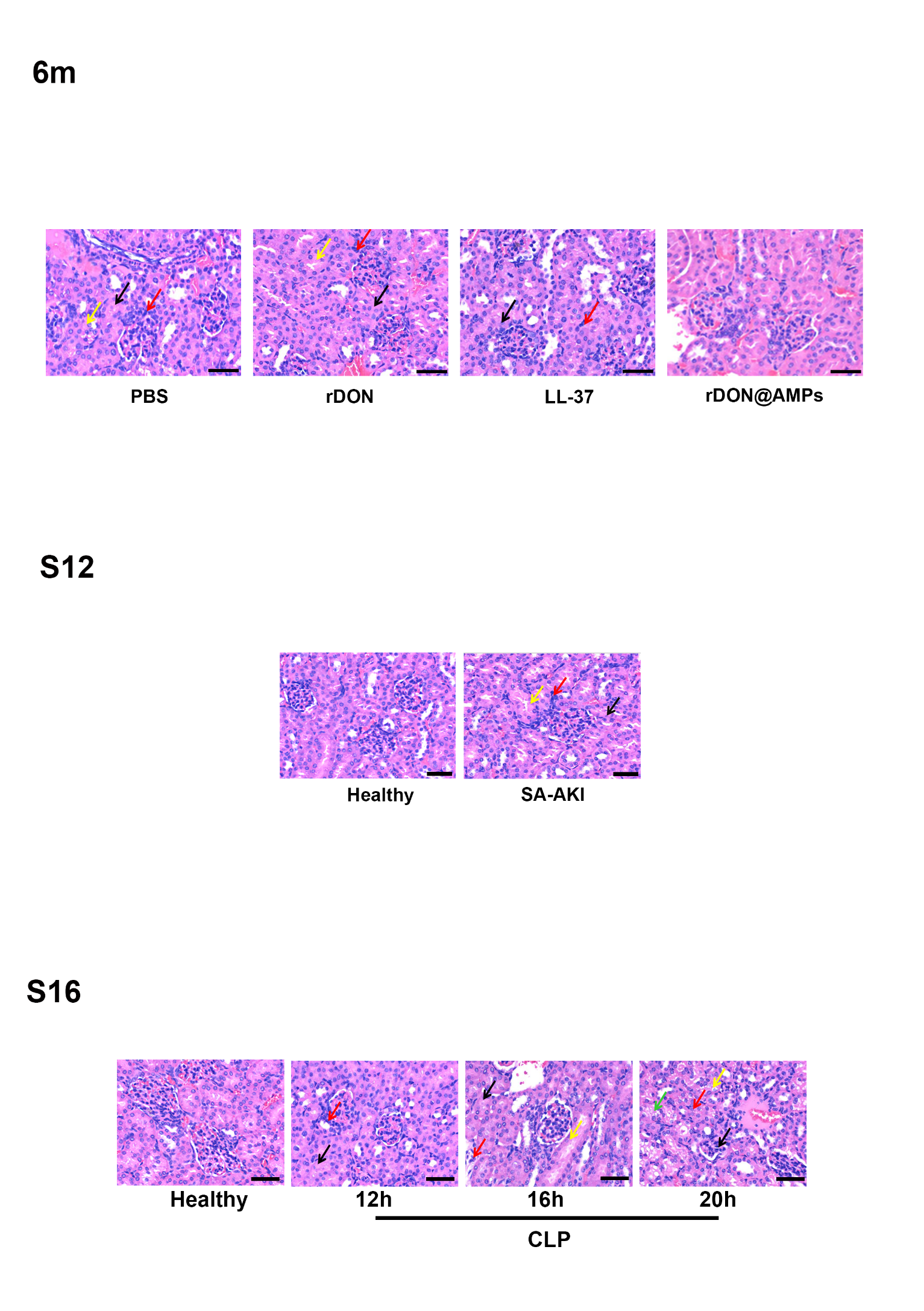


**Figure S12.** H&E staining images of healthy and SA-AKI mice. No abnormalities were observed in the healthy mice. In the SA-AKI mice, swelling of tubular epithelial cells (indicated by yellow arrows), necrosis of renal tubular epithelial cells (indicated by black arrows), and inflammatory cell infiltration (indicated by red arrows) were observed. Scale bars = 40 µm. All imaging experiments were repeated three times, yielding similar results.


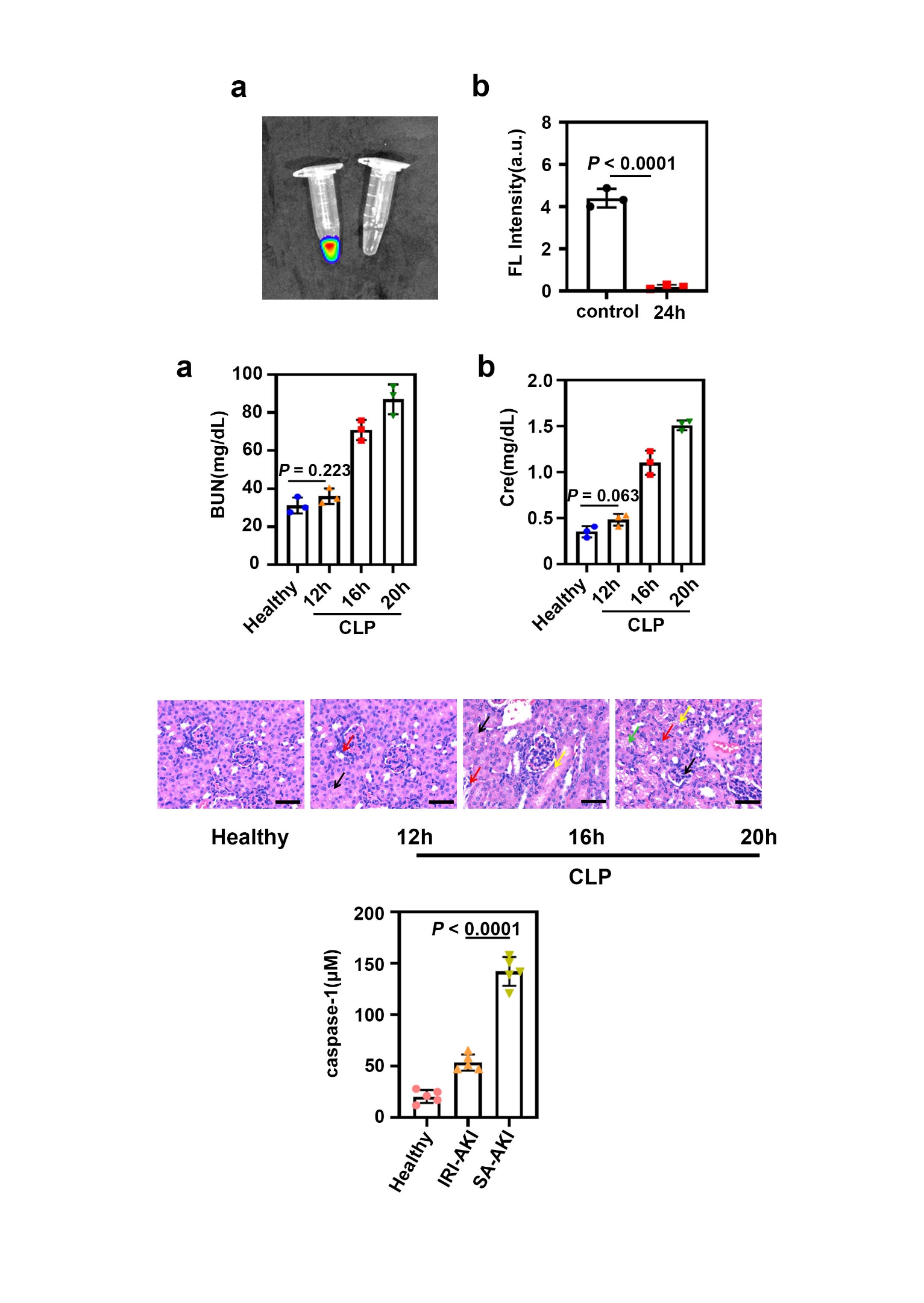


**Figure S13**. Renal clearance properties of CB-rDON@AMPs. (a) Urine from a healthy mouse was collected 24 hours after an intravenous injection of CB-rDON@AMPs, with clean urine used as a control sample. (b) Corresponding quantification of the fluorescence intensity in the urine of the healthy mouse and the clean urine collected 24 hours after the intravenous injection of CB-rDON@AMPs is presented. All imaging experiments were repeated three times, yielding similar results. Error bars represent the standard deviation obtained from three independent measurements. Statistical analysis was performed using one-way ANOVA.


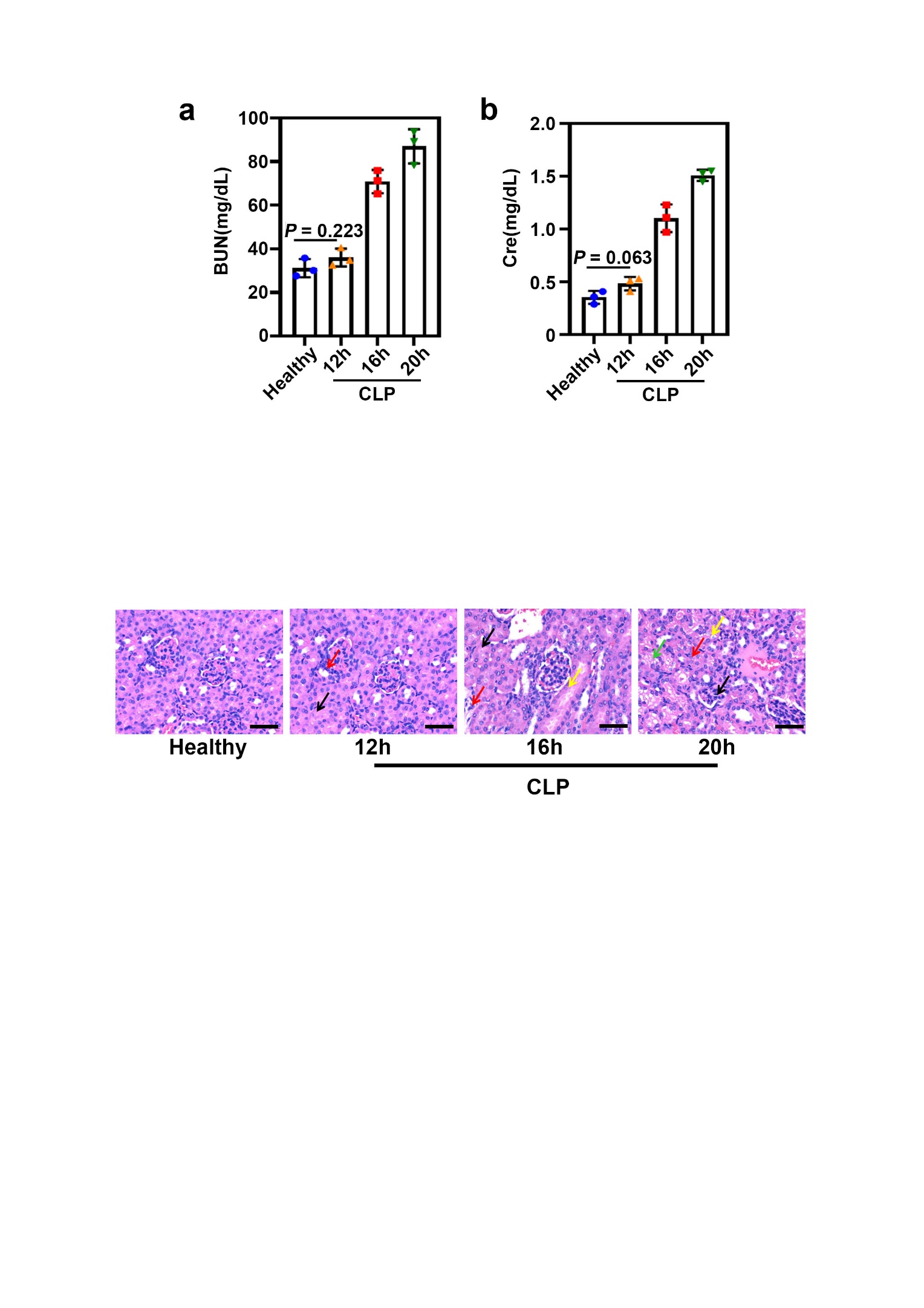


**Figure S14.** Serum biochemical parameters of mice with different CLP-treated times. (a) Blood urea nitrogen (BUN) concentration. (b) Serum creatinine(Cre) concentration. Error bars represent the standard deviation obtained from three independent measurements. Statistical analysis was performed using one-way ANOVA.


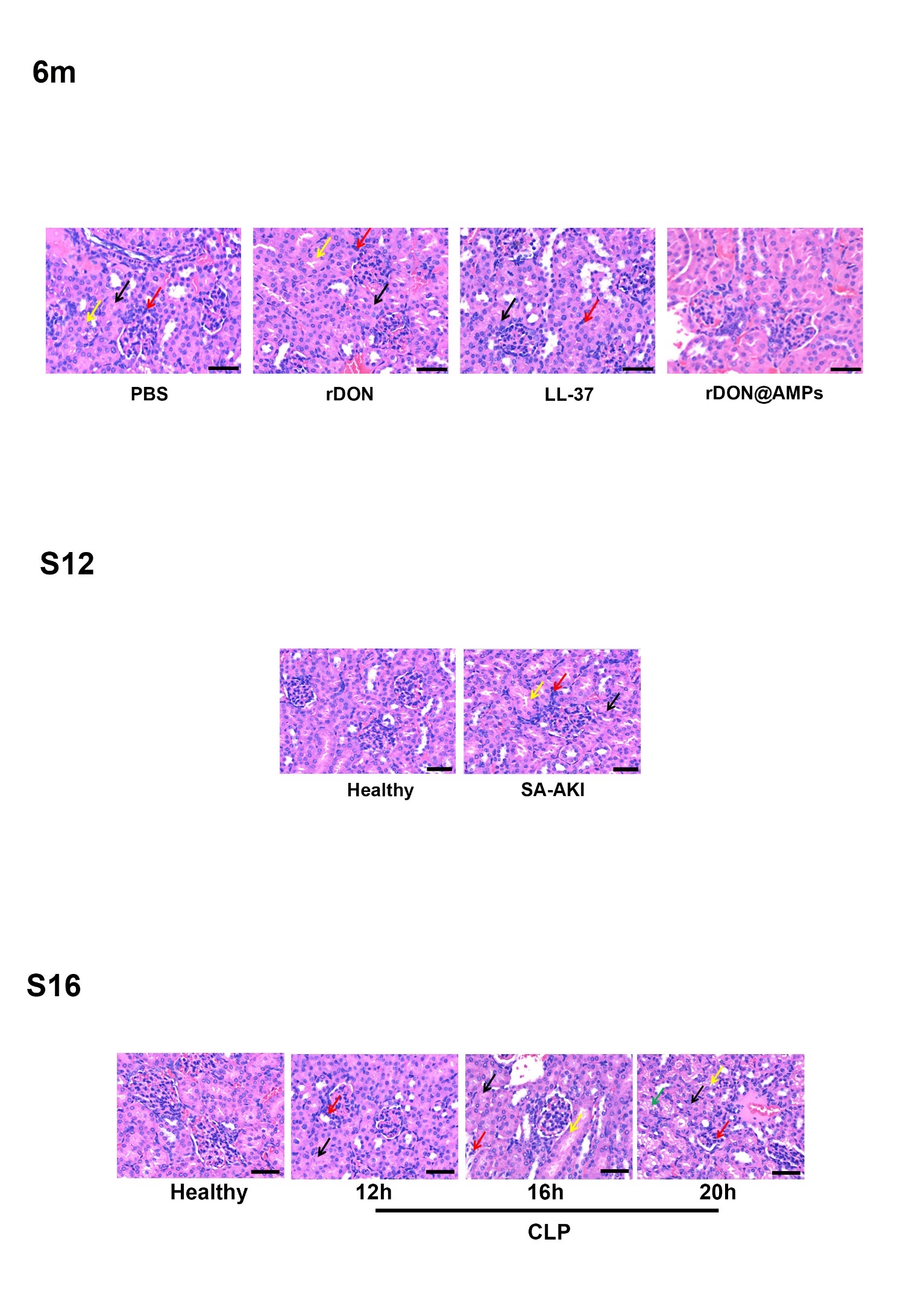


**Figure S15.**  H&E staining images of mice subjected to varying durations of CLP treatment. Observed pathological changes include inflammatory cell infiltration (red arrows), necrosis of renal tubular epithelial cells (black arrows), swelling of tubular epithelial cells (yellow arrows), and vacuolar degeneration of renal tubular epithelial cells (green arrows). Scale bars = 40 μm. All imaging experiments were repeated three times, yielding similar results.


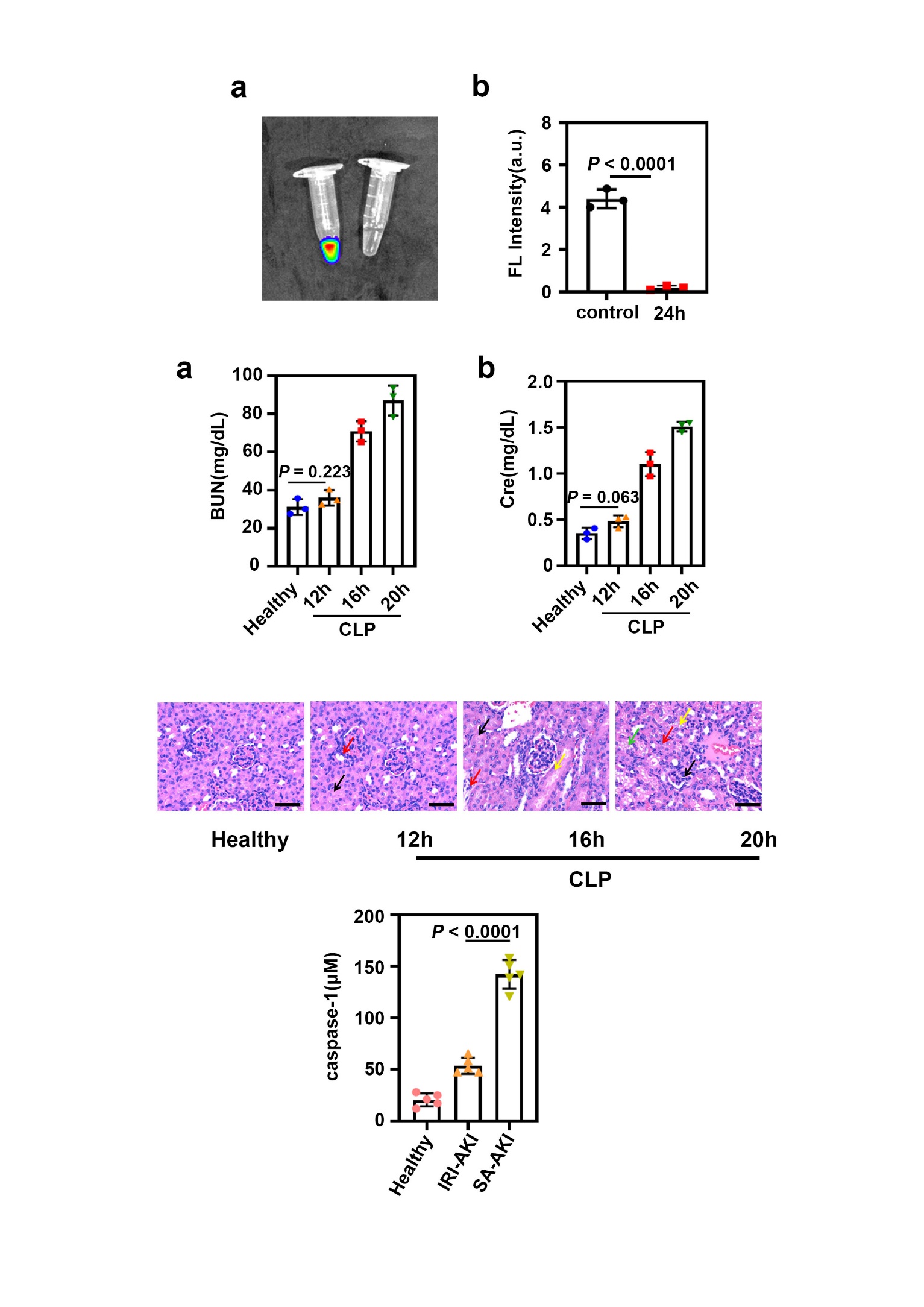


**Figure S16.** Caspase-1 enzyme activity in the kidneys of mice subjected to different treatments. Caspase-1 concentrations in the kidneys were compared among the different groups. Error bars represent the standard deviation derived from five independent measurements. Statistical analysis was performed using one-way ANOVA.

**
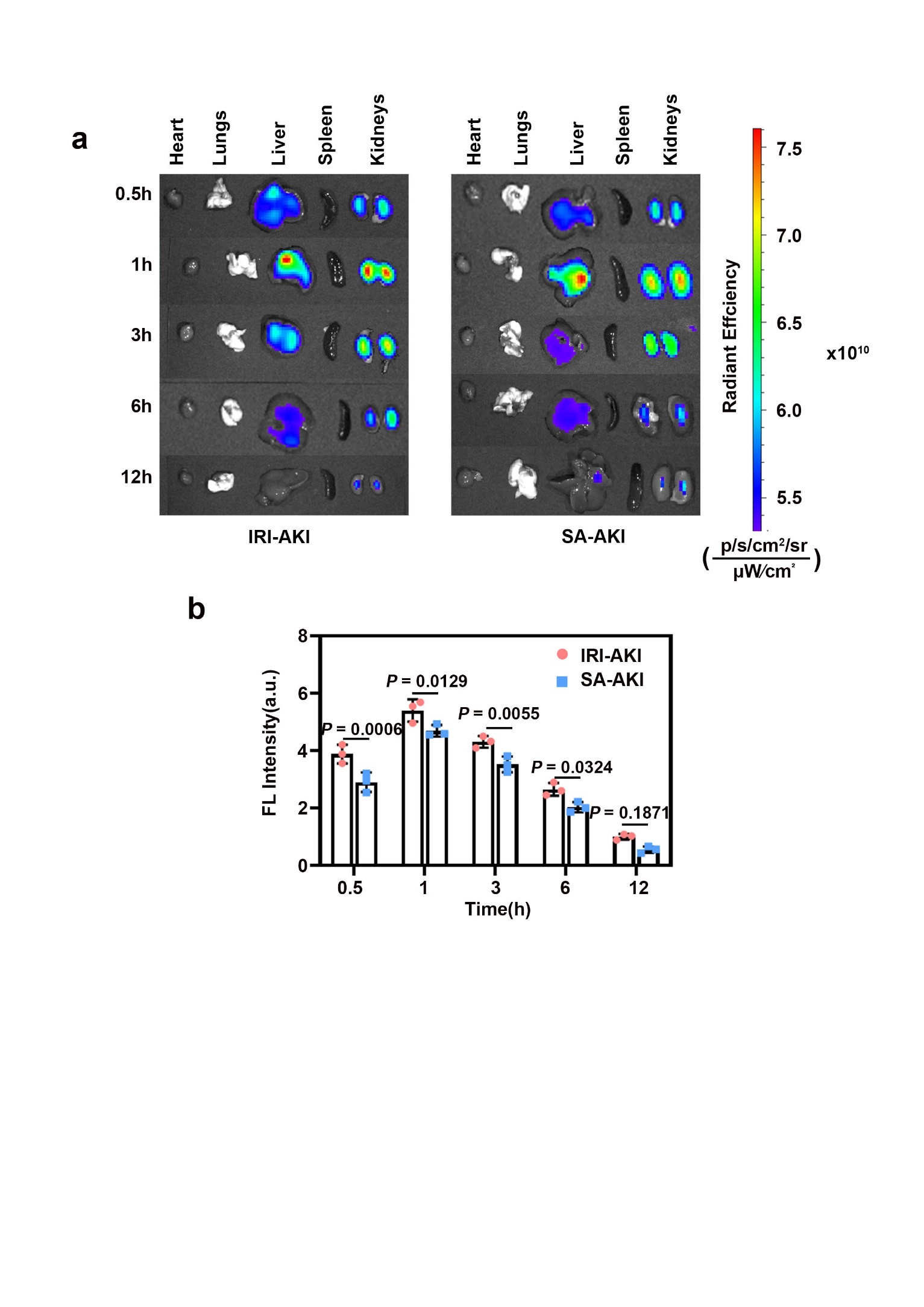
**

**Figure S17.** Ex vivo fluorescence imaging of mice with AKI induced by different etiologies. (a) Fluorescence signals of FITC were observed in major organs, including the heart, lungs, spleen, liver, and kidneys, at 0.5, 1, 3, 6, and 12 hours post-injection. (b) The corresponding quantification of fluorescence intensity is provided. All imaging experiments were repeated three times, yielding similar results. Error bars represent the standard deviation derived from three independent measurements. Statistical analysis was performed using two-way ANOVA.

**
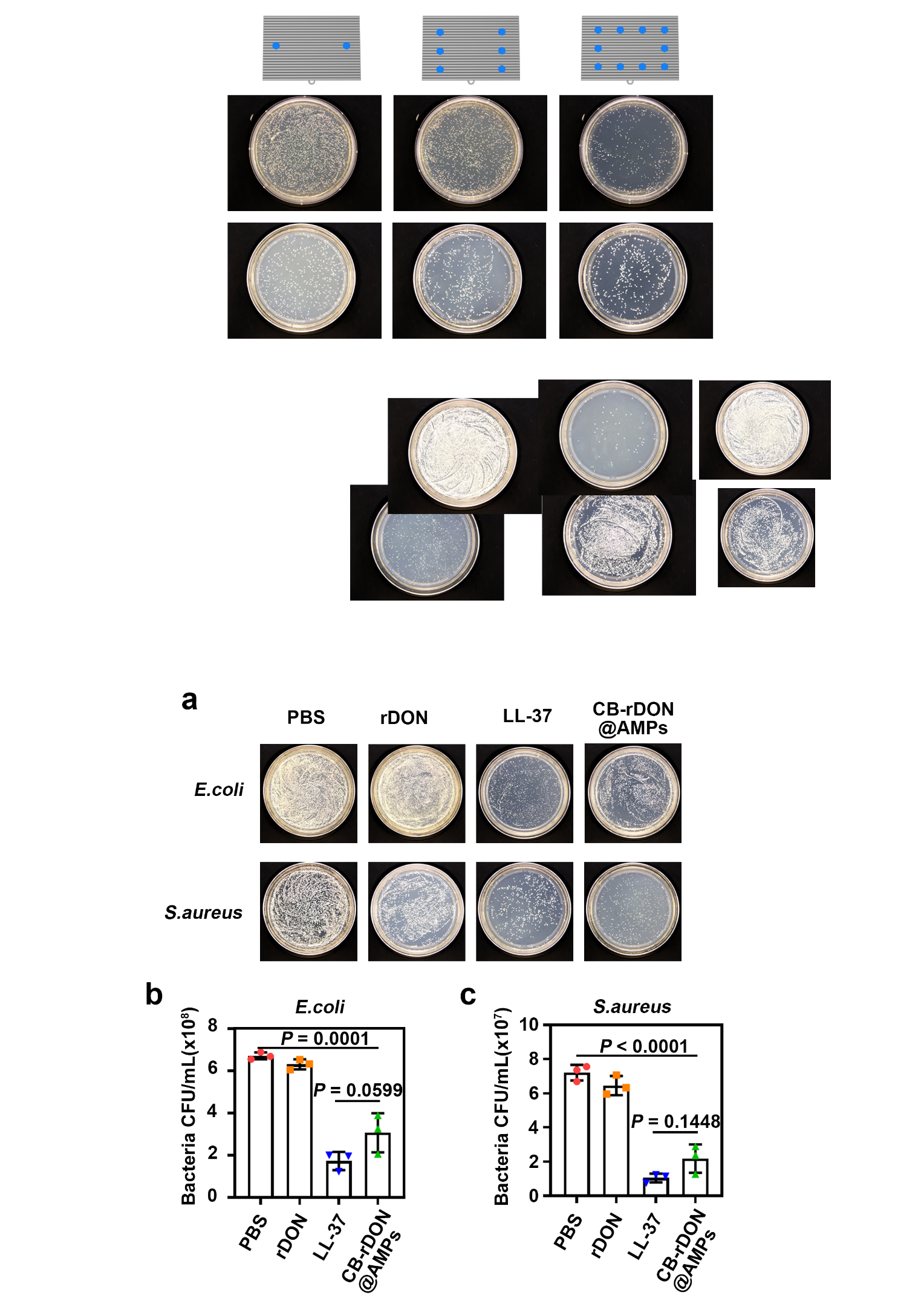
**

**Figure S18.** Antimicrobial efficacy of CB-rDON@AMPs in vitro. (a) Photographs of bacterial colonies grown from *E. coli* and *S. aureus* cultures treated with various formulations. Quantitative analysis of *E. coli* (b) and *S. aureus* (c) bacterial colony counts for the corresponding treatment groups. All imaging experiments were repeated three times, yielding similar results. Error bars represent the standard deviation derived from three independent measurements. Statistical analysis was performed using one-way ANOVA.


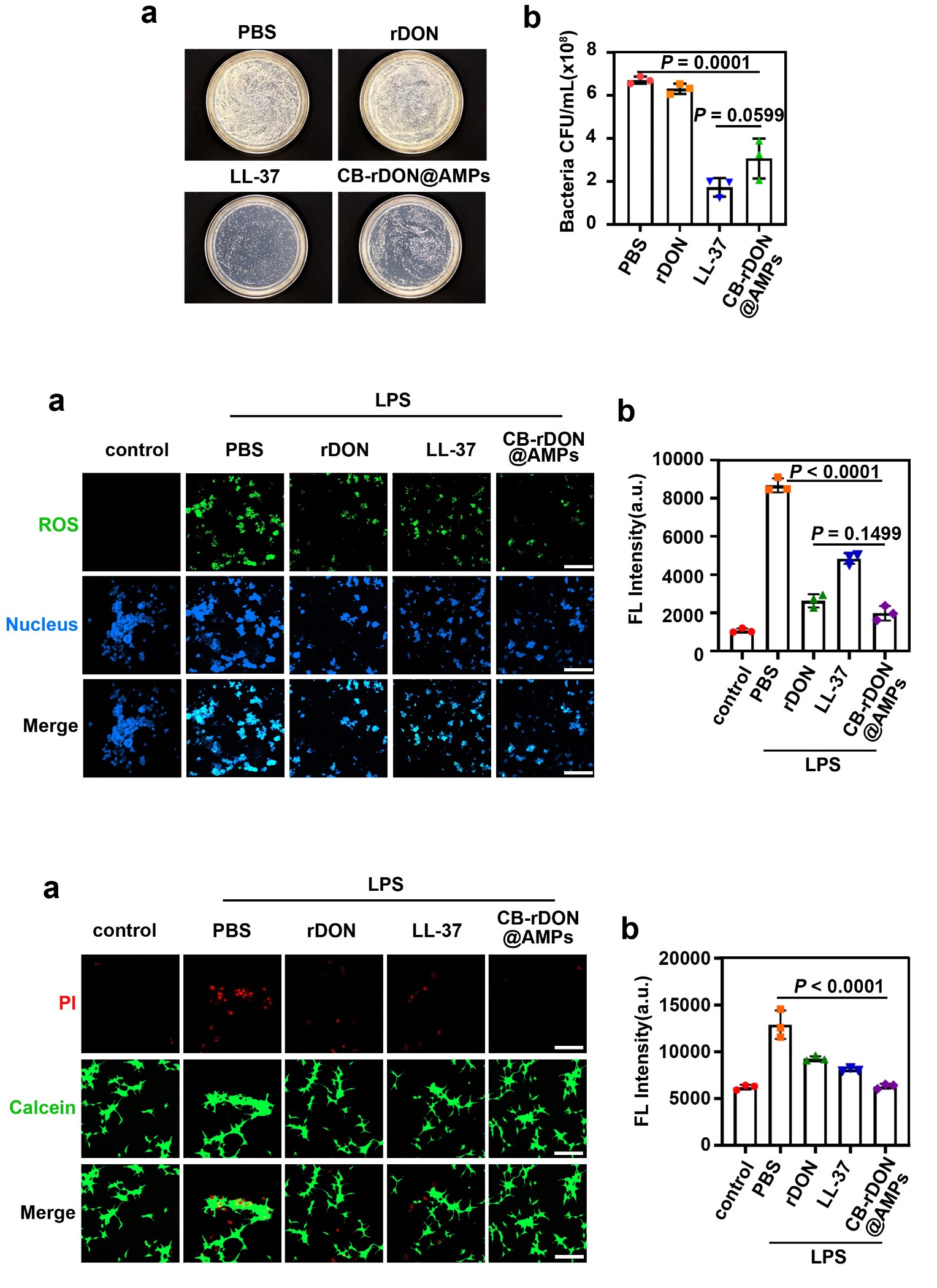


**Figure S19.** Antioxidant efficacy of CB-rDON@AMPs in vitro. Representative ROS staining (a) and corresponding quantification of ROS levels (b) in HEK-293 cells under identical treatment conditions. Scale bars = 50 μm. All imaging experiments were repeated three times, yielding similar results. Error bars represent the standard deviation derived from three independent measurements. Statistical analysis was performed using one-way ANOVA.


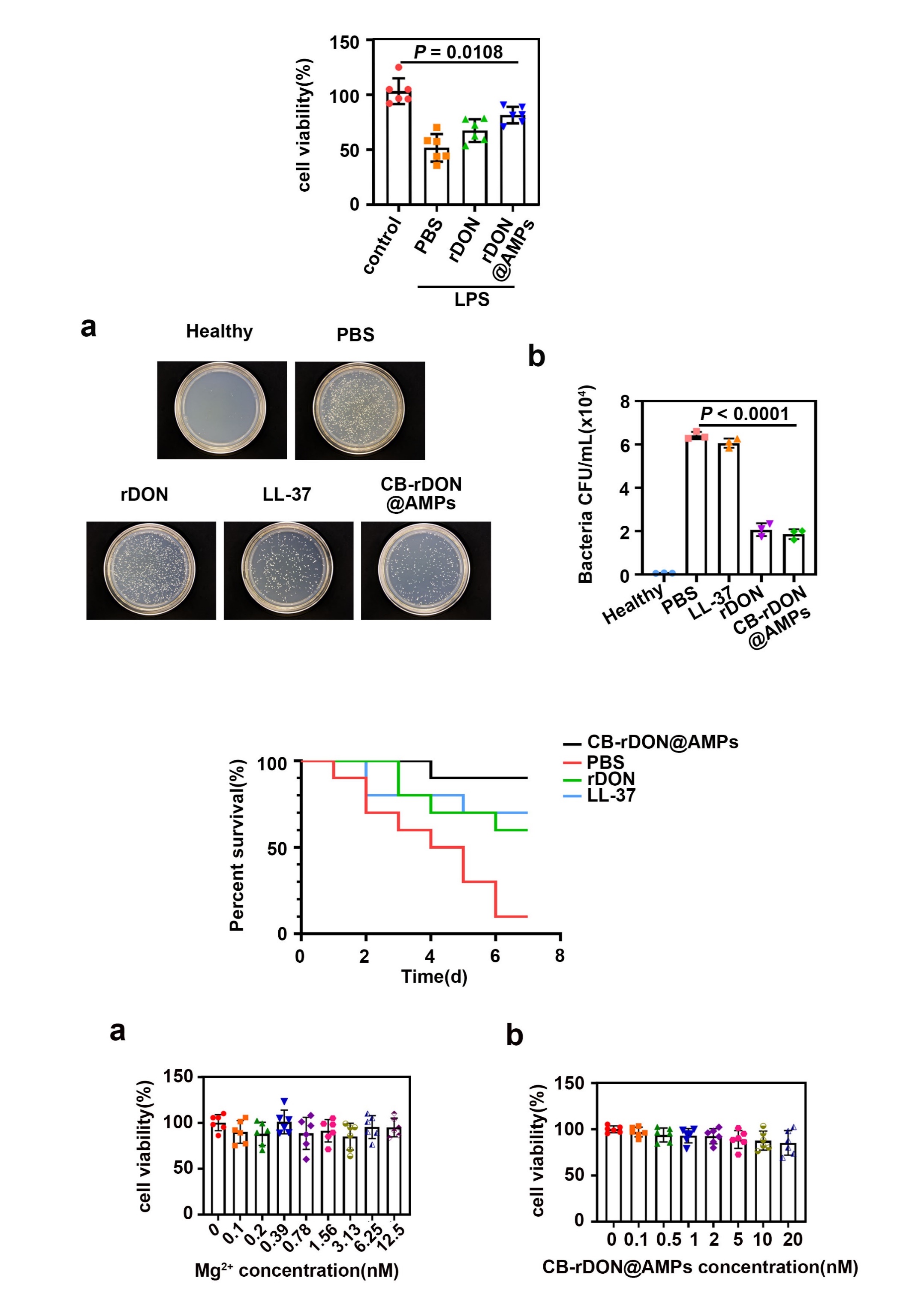


**Figure S20.**  Cellular-level evaluation of the therapeutic efficacy of CB-rDON@AMPs. A cell viability assay was conducted on HEK-293 cells under different treatment conditions. Error bars represent the standard deviation derived from six independent measurements. Statistical analysis was performed using one-way ANOVA.


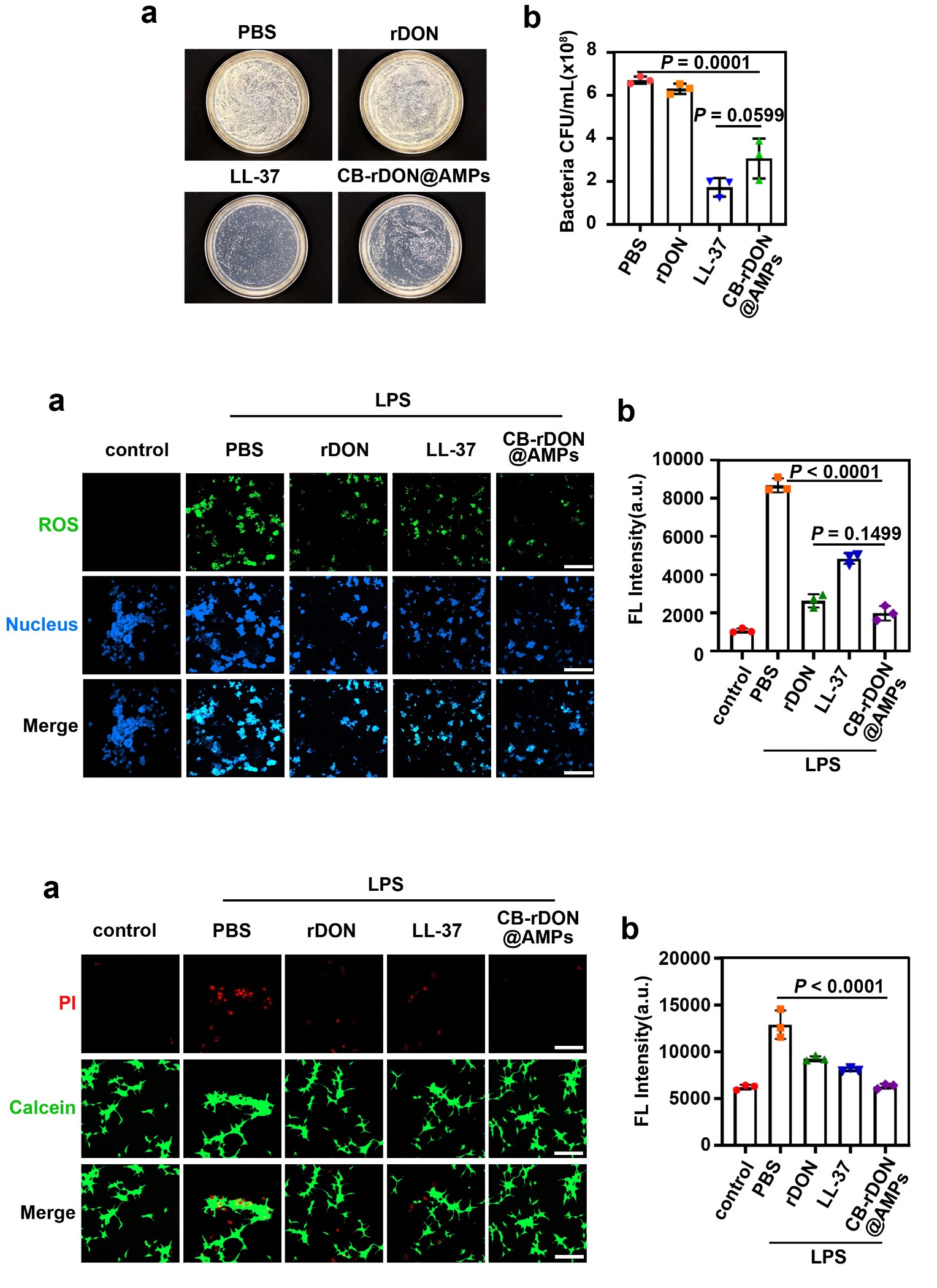


**Figure S21.** Therapeutic efficacy of CB-rDON@AMPs at the cellular level. (a) Confocal microscopy images showing live/dead staining of HEK-293 cells exposed to lipopolysaccharide (LPS, 10 μg/mL) and either PBS (100 μL), rDON (20 nM, 100 μL), LL-37 (200 nM, 100 μL), or CB-rDON@AMPs (20 nM, 100 μL). (b) Quantification of fluorescence intensity from live/dead staining assays. Scale bars = 50 μm. All imaging experiments were repeated three times, yielding similar results. Error bars represent the standard deviation derived from three independent measurements. Statistical analysis was performed using one-way ANOVA.


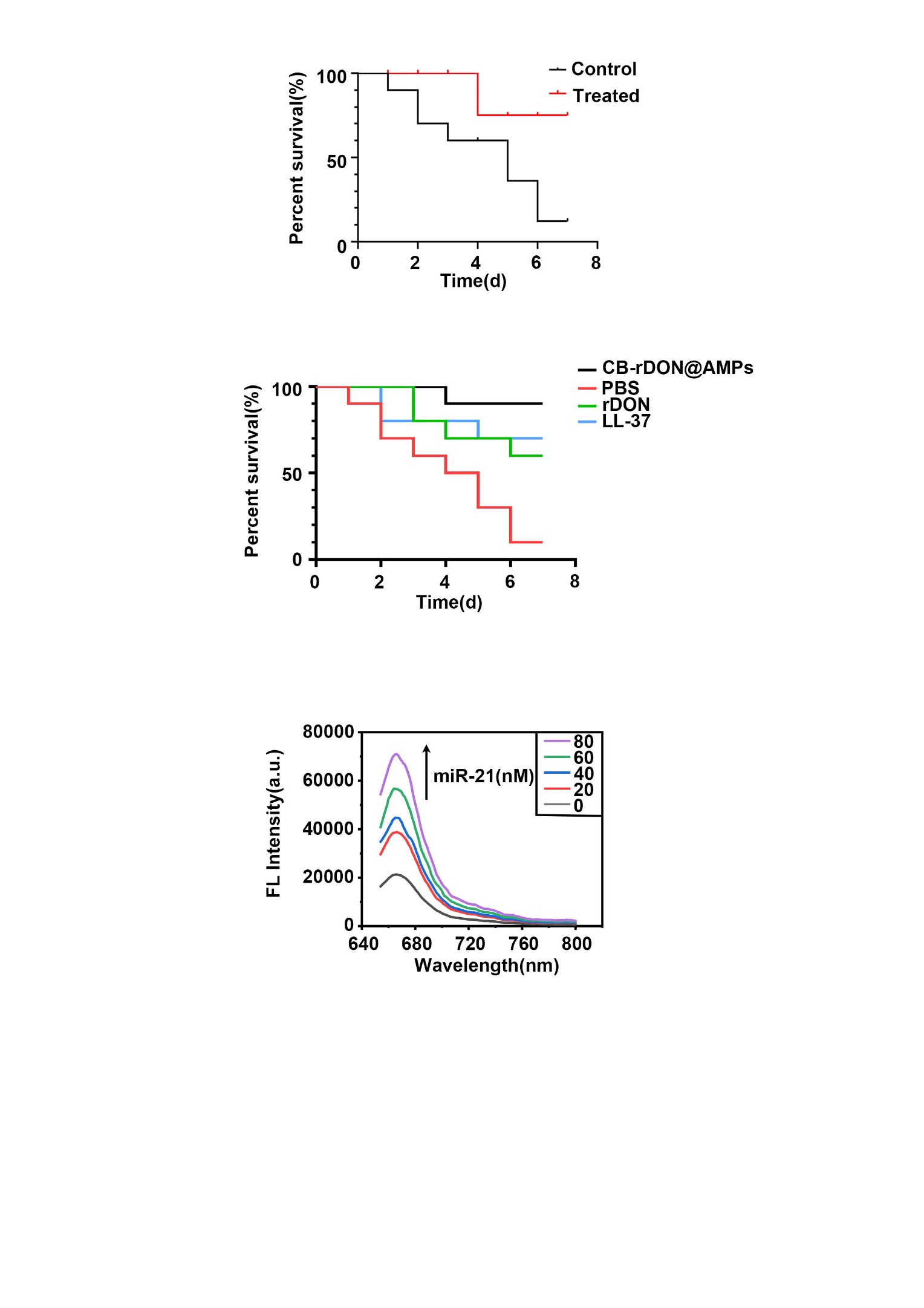


**Figure S22.** Survival curves. Seven-day survival curves of SA-AKI mice with different treatments.

**
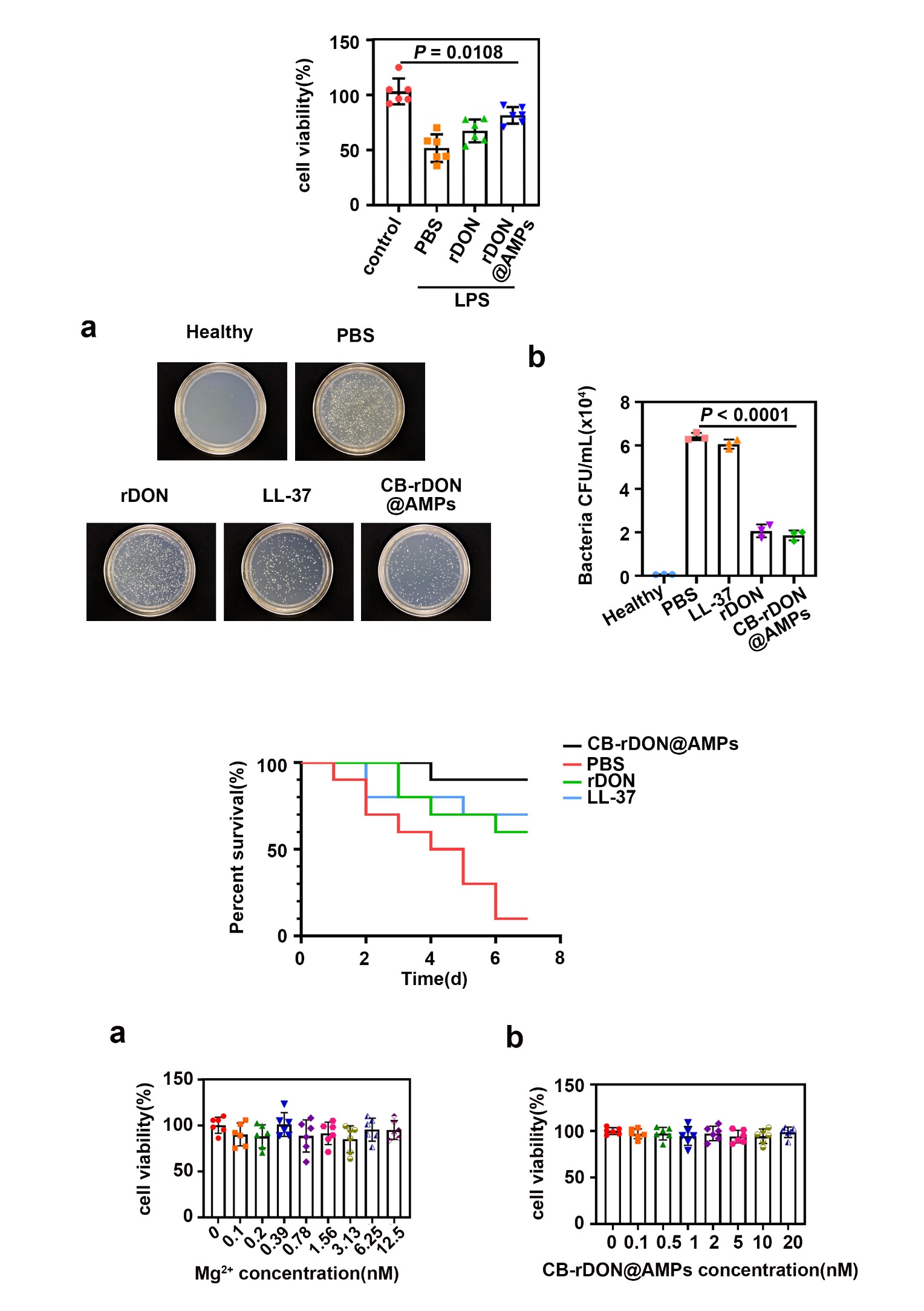
**

**Figure S23.** Cytotoxicity Test: (a) A cell viability assay was performed using HEK-293 cells incubated with varying concentrations of Mg^2+^. (b) A cell viability assay was conducted using HEK-293 cells incubated with different concentrations of CB-rDON@AMPs. Error bars represent the standard deviation derived from six independent measurements.


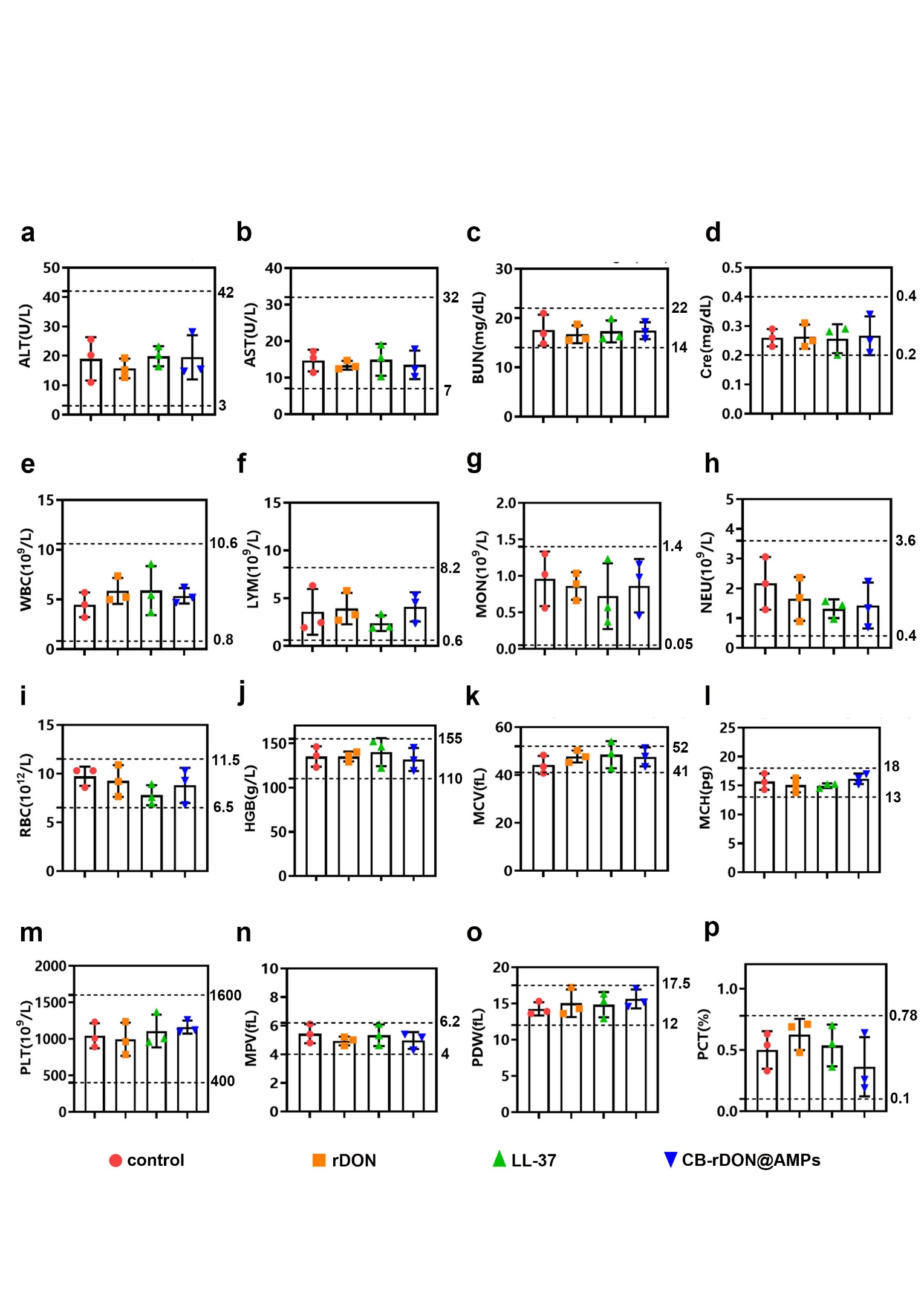


**Figure S24.**  Blood Analysis: Dashed lines indicate the normal values and ranges of parameters for healthy mice. Blood was collected 24 hours after injection. Serum levels of (a) alanine transaminase (ALT), (b) aspartate transaminase (AST), (c) blood urea nitrogen (BUN), and (d) creatinine (sCr) were measured. The numbers of (e) white blood cells (WBC), (f) lymphocytes (LYM), (g) monocytes (MON), and (h) neutrophils (NEU) were determined. The counts of (i) red blood cells (RBC), (j) hemoglobin (HGB), (k) mean corpuscular volume (MCV), and (l) mean corpuscular hemoglobin (MCH) were also assessed. Additionally, the numbers of (m) platelets (PLT), (n) mean platelet volume (MPV), (o) platelet distribution width (PDW), and (p) thrombocytocrit (PCT) were recorded. Error bars represent the standard deviation derived from three independent measurements.


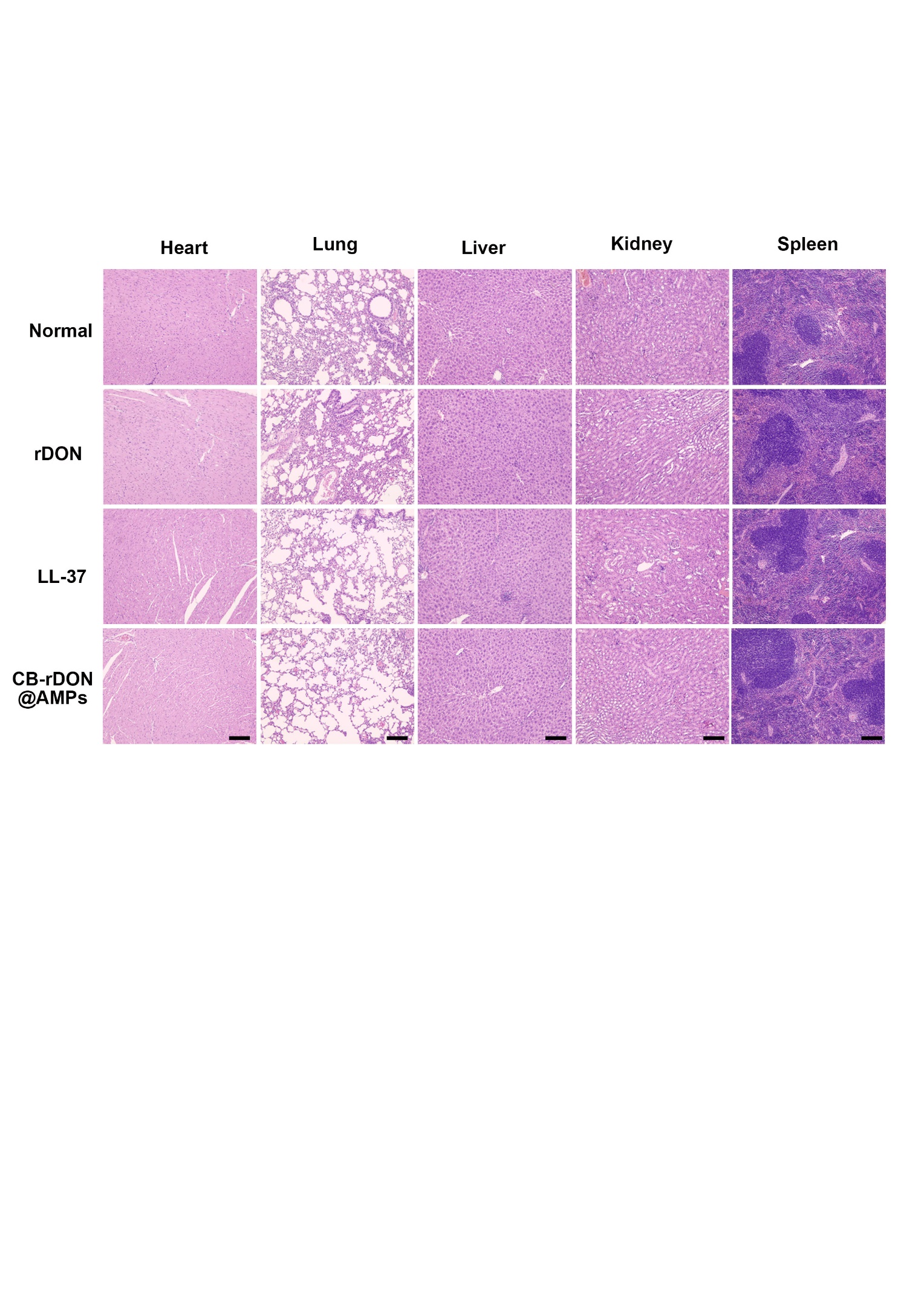


**Figure S25.** H&E staining images. Representative H&E staining images of the primary organs of mice injected with rDON , LL-37, and CB-rDON@AMPs. Tissues from healthy mice were used as controls. No abnormalities were observed. All imaging experiments were repeated three times with similar results. Scale bars = 100μm. All imaging experiments were repeated three times, yielding similar results.

**Table S1. Rectangular DNA origami tile and the corresponding staple strands (left to right 5’- 3’).**

| Name | DNA Sequence |
| --- | --- |
| 1 | CAAGCCCAATAGGAAC CCATGTACAAACAGTT |
| 2 | AATGCCCCGTAACAGT GCCCGTATCTCCCTCA |
| 3 | TGCCTTGACTGCCTAT TTCGGAACAGGGATAG |
| 4 | GAGCCGCCCCACCACC GGAACCGCGACGGAAA |
| 5 | AACCAGAGACCCTCAG AACCGCCAGGGGTCAG |
| 6 | TTATTCATAGGGAAGG TAAATATT CATTCAGT |
| 7 | CATAACCCGAGGCATA GTAAGAGC TTTTTAAG |
| 8 | ATTGAGGGTAAAGGTG AATTATCAATCACCGG |
| 9 | AAAAGTAATATCTTAC CGAAGCCCTTCCAGAG |
| 10 | GCAATAGCGCAGATAG CCGAACAATTCAACCG |
| 11 | CCTAATTTACGCTAAC GAGCGTCTAATCAATA |
| 12 | TCTTACCAGCCAGTTA CAAAATAAATGAAATA |
| 13 | ATCGGCTGCGAGCATG TAGAAACCTATCATAT |
| 14 | CTAATTTATCTTTCCT TATCATTCATCCTGAA |
| 15 | GCGTTATAGAAAAAGC CTGTTTAG AAGGCCGG |
| 16 | GCTCATTTTCGCATTA AATTTTTG AGCTTAGA |
| 17 | AATTACTACAAATTCT TACCAGTAATCCCATC |
| 18 | TTAAGACGTTGAAAAC ATAGCGATAACAGTAC |
| 19 | TAGAATCCCTGAGAAG AGTCAATAGGAATCAT |
| 20 | CTTTTACACAGATGAA TATACAGTAAACAATT |
| 21 | TTTAACGTTCGGGAGA AACAATAATTTTCCCT |
| 22 | CGACAACTAAGTATTA GACTTTACAATACCGA |
| 23 | GGATTTAGCGTATTAA ATCCTTTGTTTTCAGG |
| 24 | ACGAACCAAAACATCG CCATTAAA TGGTGGTT |
| 25 | GAACGTGGCGAGAAAG GAAGGGAA CAAACTAT |
| 26 | TAGCCCTACCAGCAGA AGATAAAAACATTTGA |
| 27 | CGGCCTTGCTGGTAAT ATCCAGAACGAACTGA |
| 28 | CTCAGAGCCACCACCC TCATTTTCCTATTATT |
| 29 | CTGAAACAGGTAATAA GTTTTAACCCCTCAGA |
| 30 | AGTGTACTTGAAAGTA TTAAGAGGCCGCCACC |
| 31 | GCCACCACTCTTTTCA TAATCAAACCGTCACC |
| 32 | GTTTGCCACCTCAGAG CCGCCACCGATACAGG |
| 33 | GACTTGAGAGACAAAA GGGCGACAAGTTACCA |
| 34 | AGCGCCAACCATTTGG GAATTAGATTATTAGC |
| 35 | GAAGGAAAATAAGAGC AAGAAACAACAGCCAT |
| 36 | GCCCAATACCGAGGAA ACGCAATAGGTTTACC |
| 37 | ATTATTTAACCCAGCT ACAATTTTCAAGAACG |
| 38 | TATTTTGCTCCCAATC CAAATAAGTGAGTTAA |
| 39 | GGTATTAAGAACAAGA AAAATAATTAAAGCCA |
| 40 | TAAGTCCTACCAAGTA CCGCACTCTTAGTTGC |
| 41 | ACGCTCAAAATAAGAA TAAACACCGTGAATTT |
| 42 | AGGCGTTACAGTAGGG CTTAATTGACAATAGA |
| 43 | ATCAAAATCGTCGCTA TTAATTAACGGATTCG |
| 44 | CTGTAAATCATAGGTC TGAGAGACGATAAATA |
| 45 | CCTGATTGAAAGAAAT TGCGTAGACCCGAACG |
| 46 | ACAGAAATCTTTGAAT ACCAAGTTCCTTGCTT |
| 47 | TTATTAATGCCGTCAA TAGATAATCAGAGGTG |
| 48 | AGATTAGATTTAAAAG TTTGAGTACACGTAAA |
| 49 | AGGCGGTCATTAGTCT TTAATGCGCAATATTA |
| 50 | GAATGGCTAGTATTAA CACCGCCTCAACTAAT |
| 51 | CCGCCAGCCATTGCAA CAGGAAAAATATTTTT |
| 52 | CCCTCAGAACCGCCAC CCTCAGAACTGAGACT |
| 53 | CCTCAAGAATACATGG CTTTTGATAGAACCAC |
| 54 | TAAGCGTCGAAGGATT AGGATTAGTACCGCCA |
| 55 | CACCAGAGTTCGGTCA TAGCCCCCGCCAGCAA |
| 56 | TCGGCATTCCGCCGCC AGCATTGACGTTCCAG |
| 57 | AATCACCAAATAGAAA ATTCATATATAACGGA |
| 58 | TCACAATCGTAGCACC ATTACCATCGTTTTCA |
| 59 | ATACCCAAGATAACCC ACAAGAATAAACGATT |
| 60 | ATCAGAGAAAGAACTG GCATGATTTTATTTTG |
| 61 | TTTTGTTTAAGCCTTA AATCAAGAATCGAGAA |
| 62 | AGGTTTTGAACGTCAA AAATGAAAGCGCTAAT |
| 63 | CAAGCAAGACGCGCCT GTTTATCAAGAATCGC |
| 64 | AATGCAGACCGTTTTT ATTTTCATCTTGCGGG |
| 65 | CATATTTAGAAATACC GACCGTGTTACCTTTT |
| 66 | AATGGTTTACAACGCC AACATGTAGTTCAGCT |
| 67 | TAACCTCCATATGTGA GTGAATAAACAAAATC |
| 68 | AAATCAATGGCTTAGG TTGGGTTACTAAATTT |
| 69 | GCGCAGAGATATCAAA ATTATTTGACATTATC |
| 70 | AACCTACCGCGAATTA TTCATTTCCAGTACAT |
| 71 | ATTTTGCGTCTTTAGG AGCACTAAGCAACAGT |
| 72 | CTAAAATAGAACAAAG AAACCACCAGGGTTAG |
| 73 | GCCACGCTATACGTGG CACAGACAACGCTCAT |
| 74 | GCGTAAGAGAGAGCCA GCAGCAAAAAGGTTAT |
| 75 | GGAAATACCTACATTT TGACGCTCACCTGAAA |
| 76 | TATCACCGTACTCAGG AGGTTTAGCGGGGTTT |
| 77 | TGCTCAGTCAGTCTCT GAATTTACCAGGAGGT |
| 78 | GGAAAGCGACCAGGCG GATAAGTGAATAGGTG |
| 79 | TGAGGCAGGCGTCAGA CTGTAGCGTAGCAAGG |
| 80 | TGCCTTTAGTCAGACG ATTGGCCTGCCAGAAT |
| 81 | CCGGAAACACACCACG GAATAAGTAAGACTCC |
| 82 | ACGCAAAGGTCACCAA TGAAACCAATCAAGTT |
| 83 | TTATTACGGTCAGAGG GTAATTGAATAGCAGC |
| 84 | TGAACAAACAGTATGT TAGCAAACTAAAAGAA |
| 85 | CTTTACAGTTAGCGAA CCTCCCGACGTAGGAA |
| 86 | GAGGCGTTAGAGAATA ACATAAAAGAACACCC |
| 87 | TCATTACCCGACAATA AACAACATATTTAGGC |
| 88 | CCAGACGAGCGCCCAA TAGCAAGCAAGAACGC |
| 89 | AGAGGCATAATTTCAT CTTCTGACTATAACTA |
| 90 | TTTTAGTTTTTCGAGC CAGTAATAAATTCTGT |
| 91 | TATGTAAACCTTTTTT AATGGAAAAATTACCT |
| 92 | TTGAATTATGCTGATG CAAATCCACAAATATA |
| 93 | GAGCAAAAACTTCTGA ATAATGGAAGAAGGAG |
| 94 | TGGATTATGAAGATGA TGAAACAAAATTTCAT |
| 95 | CGGAATTATTGAAAGG AATTGAGGTGAAAAAT |
| 96 | ATCAACAGTCATCATA TTCCTGATTGATTGTT |
| 97 | CTAAAGCAAGATAGAA CCCTTCTGAATCGTCT |
| 98 | GCCAACAGTCACCTTG CTGAACCTGTTGGCAA |
| 99 | GAAATGGATTATTTAC ATTGGCAGACATTCTG |
| 100 | TTTT TATAAGTA TAGCCCGGCCGTCGAG |
| 101 | AGGGTTGA TTTT ATAAATCC |
| 102 | ACAAACAA TTTT AATCAGTA GCGACAGATCGATAGC |
| 103 | AGCACCGT TTTT TAAAGGTG GCAACATAGTAGAAAA |
| 104 | TACATACA TTTT GACGGGAG |
| 105 | GCGCATTA TTTT GCTTATCC |
| 106 | TATAGAAG TTTT CGACAAAA GGTAAAGTAGAGAATA |
| 107 | TAAAGTAC TTTT CGCGAGAA AACTTTTTATCGCAAG |
| 108 | ACAAAGAA TTTT ATTAATTA CATTTAACACATCAAG |
| 109 | AAAACAAA TTTT TTCATCAA TATAATCCTATCAGAT |
| 110 | GATGGCAA TTTT AATCAATA TCTGGTCACAAATATC |
| 111 | AAACCCTC TTTT ACCAGTAA TAAAAGGGATTCACCA GTCACACG TTTT |
| 112 | CCGAAATCCGAAAATC CTGTTTGAAGCCGGAA |
| 113 | CCAGCAGGGGCAAAAT CCCTTATAAAGCCGGC |
| 114 | GCATAAAGTTCCACAC AACATACGAAGCGCCA |
| 115 | GCTCACAATGTAAAGC CTGGGGTGGGTTTGCC |
| 116 | TTCGCCATTGCCGGAA ACCAGGCATTAAATCA |
| 117 | GCTTCTGGTCAGGCTG CGCAACTGTGTTATCC |
| 118 | GTTAAAATTTTAACCA ATAGGAACCCGGCACC |
| 119 | AGACAGTCATTCAAAA GGGTGAGAAGCTATAT |
| 120 | AGGTAAAGAAATCACC ATCAATATAATATTTT |
| 121 | TTTCATTTGGTCAATA ACCTGTTTATATCGCG |
| 122 | TCGCAAATGGGGCGCG AGCTGAAATAATGTGT |
| 123 | TTTTAATTGCCCGAAA GACTTCAAAACACTAT |
| 124 | AAGAGGAACGAGCTTC AAAGCGAAGATACATT |
| 125 | GGAATTACTCGTTTAC CAGACGACAAAAGATT |
| 126 | GAATAAGGACGTAACA AAGCTGCTCTAAAACA |
| 127 | CCAAATCACTTGCCCT GACGAGAACGCCAAAA |
| 128 | CTCATCTTGAGGCAAA AGAATACAGTGAATTT |
| 129 | AAACGAAATGACCCCC AGCGATTATTCATTAC |
| 130 | CTTAAACATCAGCTTG CTTTCGAGCGTAACAC |
| 131 | TCGGTTTAGCTTGATA CCGATAGTCCAACCTA |
| 132 | TGAGTTTCGTCACCAG TACAAACTTAATTGTA |
| 133 | CCCCGATTTAGAGCTT GACGGGGAAATCAAAA |
| 134 | GAATAGCCGCAAGCGG TCCACGCTCCTAATGA |
| 135 | GAGTTGCACGAGATAG GGTTGAGTAAGGGAGC |
| 136 | GTGAGCTAGTTTCCTG TGTGAAATTTGGGAAG |
| 137 | TCATAGCTACTCACAT TAATTGCGCCCTGAGA |
| 138 | GGCGATCGCACTCCAG CCAGCTTTGCCATCAA |
| 139 | GAAGATCGGTGCGGGC CTCTTCGCAATCATGG |
| 140 | AAATAATTTTAAATTG TAAACGTTGATATTCA |
| 141 | GCAAATATCGCGTCTG GCCTTCCTGGCCTCAG |
| 142 | ACCGTTCTAAATGCAA TGCCTGAGAGGTGGCA |
| 143 | TATATTTTAGCTGATA AATTAATGTTGTATAA |
| 144 | TCAATTCTTTTAGTTT GACCATTACCAGACCG |
| 145 | CGAGTAGAACTAATAG TAGTAGCAAACCCTCA |
| 146 | GAAGCAAAAAAGCGGA TTGCATCAGATAAAAA |
| 147 | TCAGAAGCCTCCAACA GGTCAGGATCTGCGAA |
| 148 | CCAAAATATAATGCAG ATACATAAACACCAGA |
| 149 | CATTCAACGCGAGAGG CTTTTGCATATTATAG |
| 150 | ACGAGTAGTGACAAGA ACCGGATATACCAAGC |
| 151 | AGTAATCTTAAATTGG GCTTGAGAGAATACCA |
| 152 | GCGAAACATGCCACTA CGAAGGCATGCGCCGA |
| 153 | ATACGTAAAAGTACAA CGGAGATTTCATCAAG |
| 154 | CAATGACACTCCAAAA GGAGCCTTACAACGCC |
| 155 | AAAAAAGGACAACCAT CGCCCACGCGGGTAAA |
| 156 | TGTAGCATTCCACAGA CAGCCCTCATCTCCAA |
| 157 | GTAAAGCACTAAATCG GAACCCTAGTTGTTCC |
| 158 | AGTTTGGAGCCCTTCA CCGCCTGGTTGCGCTC |
| 159 | AGCTGATTACAAGAGT CCACTATTGAGGTGCC |
| 160 | ACTGCCCGCCGAGCTC GAATTCGTTATTACGC |
| 161 | CCCGGGTACTTTCCAG TCGGGAAACGGGCAAC |
| 162 | CAGCTGGCGGACGACG ACAGTATCGTAGCCAG |
| 163 | GTTTGAGGGAAAGGGG GATGTGCTAGAGGATC |
| 164 | CTTTCATCCCCAAAAA CAGGAAGACCGGAGAG |
| 165 | AGAAAAGCAACATTAA ATGTGAGCATCTGCCA |
| 166 | GGTAGCTAGGATAAAA ATTTTTAGTTAACATC |
| 167 | CAACGCAATTTTTGAG AGATCTACTGATAATC |
| 168 | CAATAAATACAGTTGA TTCCCAATTTAGAGAG |
| 169 | TCCATATACATACAGG CAAGGCAACTTTATTT |
| 170 | TACCTTTAAGGTCTTT ACCCTGACAAAGAAGT |
| 171 | CAAAAATCATTGCTCC TTTTGATAAGTTTCAT |
| 172 | TTTGCCAGATCAGTTG AGATTTAGTGGTTTAA |
| 173 | AAAGATTCAGGGGGTA ATAGTAAACCATAAAT |
| 174 | TTTCAACTATAGGCTG GCTGACCTTGTATCAT |
| 175 | CCAGGCGCTTAATCAT TGTGAATTACAGGTAG |
| 176 | CGCCTGATGGAAGTTT CCATTAAACATAACCG |
| 177 | TTTCATGAAAATTGTG TCGAAATCTGTACAGA |
| 178 | ATATATTCTTTTTTCA CGTTGAAAATAGTTAG |
| 179 | AATAATAAGGTCGCTG AGGCTTGCAAAGACTT |
| 180 | CGTAACGATCTAAAGT TTTGTCGTGAATTGCG |
| 181 | ACCCAAATCAAGTTTT TTGGGGTCAAAGAACG |
| 182 | TGGACTCCCTTTTCAC CAGTGAGACCTGTCGT |
| 183 | TGGTTTTTAACGTCAA AGGGCGAAGAACCATC |
| 184 | GCCAGCTGCCTGCAGG TCGACTCTGCAAGGCG |
| 185 | CTTGCATGCATTAATG AATCGGCCCGCCAGGG |
| 186 | ATTAAGTTCGCATCGT AACCGTGCGAGTAACA |
| 187 | TAGATGGGGGGTAACG CCAGGGTTGTGCCAAG |
| 188 | ACCCGTCGTCATATGT ACCCCGGTAAAGGCTA |
| 189 | CATGTCAAGATTCTCC GTGGGAACCGTTGGTG |
| 190 | TCAGGTCACTTTTGCG GGAGAAGCAGAATTAG |
| 191 | CTGTAATATTGCCTGA GAGTCTGGAAAACTAG |
| 192 | CAAAATTAAAGTACGG TGTCTGGAAGAGGTCA |
| 193 | TGCAACTAAGCAATAA AGCCTCAGTTATGACC |
| 194 | TTTTTGCGCAGAAAAC GAGAATGAATGTTTAG |
| 195 | AAACAGTTGATGGCTT AGAGCTTATTTAAATA |
| 196 | ACTGGATAACGGAACA ACATTATTACCTTATG |
| 197 | ACGAACTAGCGTCCAA TACTGCGGAATGCTTT |
| 198 | CGATTTTAGAGGACAG ATGAACGGCGCGACCT |
| 199 | CTTTGAAAAGAACTGG CTCATTATTTAATAAA |
| 200 | GCTCCATGAGAGGCTT TGAGGACTAGGGAGTT |
| 201 | ACGGCTACTTACTTAG CCGGAACGCTGACCAA |
| 202 | AAAGGCCGAAAGGAAC AACTAAAGCTTTCCAG |
| 203 | GAGAATAGCTTTTGCG GGATCGTCGGGTAGCA |
| 204 | ACGTTAGTAAATGAAT TTTCTGTAAGCGGAGT |
| 205 | TTTTCGATGGCC CACTACGTAAACCGTC |
| 206 | TATCAGGGTTTTCGGTTTGCGTATTGGGAACGCGCG |
| 207 | GGGAGAGGTTTT TGTAAAACGACGGCCATTCCCAGT |
| 208 | CACGACGTTTTTGTAATGGGATAGGTCAAAACGGCG |
| 209 | GATTGACCTTTTGATGAACGGTAATCGTAGCAAACA |
| 210 | AGAGAATCTTTTGGTTGTACCAAAAACAAGCATAAA |
| 211 | GCTAAATCTTTTCTGTAGCTCAACATGTATTGCTGA |
| 212 | ATATAATGTTTTCATTGAATCCCCCTCAAATCGTCA |
| 213 | TAAATATTTTTTGGAAGAAA AATCTACGACCAGTCA |
| 214 | GGACGTTGTTTTTCATAAGGGAACCGAAAGGCGCAG |
| 215 | ACGGTCAATTTTGACAGCATCGGAACGAACCCTCAG |
| 216 | CAGCGAAAATTTTACTTTCAACAGTTTCTGGGATTTTG  CTAAACTTTT |
| Loop1 | AACATCACTTGCCTGAGTAGAAGAACT |
| Loop2 | TGTAGCAATACTTCTTTGATTAGTAAT |
| Loop3 | AGTCTGTCCATCACGCAAATTAACCGT |
| Loop4 | ATAATCAGTGAGGCCACCGAGTAAAAG |
| Loop5 | ACGCCAGAATCCTGAGAAGTGTTTTT |
| Loop6 | TTAAAGGGATTTTAGACAGGAACGGT |
| Loop7 | AGAGCGGGAGCTAAACAGGAGGCCGA |
| Loop8 | TATAACGTGCTTTCCTCGTTAGAATC |
| Loop9 | GTACTATGGTTGCTTTGACGAGCACG |
| Loop10 | GCGCTTAATGCGCCGCTACAGGGCGC |

**Table S2. The sequence of short strands modified with Cy5. (left to right 5’- 3’)**

| Name | Sequence |
| --- | --- |
| 10’ | Cy5-TTTTTTATAGCTTTGATAGAGCAATAGCGCAGATAG CCGAACAATTCAACCG |
| 14’ | Cy5-TTTTTTATAGCTTTGATAGACTAATTTATCTTTCCT TATCATTCATCCTGAA |
| 19’ | Cy5-TTTTTTATAGCTTTGATAGATAGAATCCCTGAGAAG AGTCAATAGGAATCAT |
| 34’ | Cy5-TTTTTTATAGCTTTGATAGAAGCGCCAACCATTTGG GAATTAGATTATTAGC |
| 38’ | Cy5-TTTTTTATAGCTTTGATAGATATTTTGCTCCCAATC CAAATAAGTGAGTTAA |
| 42’ | Cy5-TTTTTTATAGCTTTGATAGAAGGCGTTACAGTAGGG CTTAATTGACAATAGA |
| 46’ | Cy5-TTTTTTATAGCTTTGATAGAACAGAAATCTTTGAAT ACCAAGTTCCTTGCTT |
| 118’ | Cy5-TTTTTTATAGCTTTGATAGAGTTAAAATTTTAACCA ATAGGAACCCGGCACC |
| 122’ | Cy5-TTTTTTATAGCTTTGATAGATCGCAAATGGGGCGCG AGCTGAAATAATGTGT |
| 125’ | Cy5-TTTTTTATAGCTTTGATAGAGGAATTACTCGTTTAC CAGACGACAAAAGATT |
| 139’ | Cy5-TTTTTTATAGCTTTGATAGAGAAGATCGGTGCGGGC CTCTTCGCAATCATGG |
| 143’ | Cy5-TTTTTTATAGCTTTGATAGATATATTTTAGCTGATA AATTAATGTTGTATAA |
| 147’ | Cy5-TTTTTTATAGCTTTGATAGATCAGAAGCCTCCAACA GGTCAGGATCTGCGAA |
| 151’ | Cy5-TTTTTTATAGCTTTGATAGAAGTAATCTTAAATTGG GCTTGAGAGAATACCA |

**Table S3. The sequence of short strands modified with Cy5. (left to right 5’- 3’)**

| Name | Sequence |
| --- | --- |
| BHQ3-ssDNA | TTTTTTCATCTATCAAGCCCATCGACTGCAGCTATAGAAGC-BHQ3 |
| miR-21 | CAACAGCAGUCGAUGGGCUGUC |

**Table S4. The sequence of capture strands. (left to right 5’- 3’)**

| Name | Sequence |
| --- | --- |
| 29-L | AAAAAAAAAAAAAAACTGAAACAGGTAATAA  GTTTTAACCCCTCAGA |
| 47-L | AAAAAAAAAAAAAAATTATTAATGCCGTCAA  TAGATAATCAGAGGTG |
| 56-L | AAAAAAAAAAAAAAATCGGCATTCCGCCGCC  AGCATTGACGTTCCAG |
| 62-L | AAAAAAAAAAAAAAAAGGTTTTGAACGTCAA  AAATGAAAGCGCTAAT |
| 72-L | AAAAAAAAAAAAAAACTAAAATAGAACAAAG  AAACCACCAGGGTTAG |
| 115-L | AAAAAAAAAAAAAAAGCTCACAATGTAAAGC  CTGGGGTGGGTTTGCC |
| 131-L | AAAAAAAAAAAAAAATCGGTTTAGCTTGATA  CCGATAGTCCAACCTA |
| 161-L | AAAAAAAAAAAAAAACCCGGGTACTTTCCAG  TCGGGAAACGGGCAAC |
| 169-L | AAAAAAAAAAAAAAATCCATATACATACAGG  CAAGGCAACTTTATTT |
| 177-L | AAAAAAAAAAAAAAATTTCATGAAAATTGTG  TCGAAATCTGTACAGA |

SH-ssDNA: SH-TTTTTTTTTTTTTTT

**Table S5. Annealing program for self-assembly of DNA origami nanostructures. Each step represents 1 ºC of temperature change.**

| **Temperature** | **TIME/STEP** |
| --- | --- |
| 90 ℃ | 30sec |
| 86-71 ℃ | 1min/step |
| 70-60 ℃ | 10min/step |
| 59-30 ℃ | 15min/step |
| 29-26 ℃ | 10min/step |
| 25 ℃ | hold |

**Table S6. The sequence of PCR primer (left to right 5’-3’)**

| Name | Sequence |
| --- | --- |
| miR-21-loop primer | GTCGTATCCAGTGCAGGGTCCGAGGTATTCGCACTGGATACGACGACAGC |
| Forward primer | GCCAACAGCAGTCGATGG |
| Reverse primer | GTGCAGGGTCCGAGGT |
| U6 Forward primer | CGCTTCGGCAGACATATAC |
| U6 Reverse primer | AAATATGGAACGCTTCACGA |
